# *Mycobacterium bovis* Population Structure in Cattle and Local Badgers: Co-Localisation and Variation by Farm Type

**DOI:** 10.3390/pathogens9070592

**Published:** 2020-07-21

**Authors:** Georgina Milne, Adrian Allen, Jordon Graham, Raymond Kirke, Carl McCormick, Eleanor Presho, Robin Skuce, Andrew W. Byrne

**Affiliations:** 1 Veterinary Sciences Division, Agri-Food and Biosciences Institute (AFBI), Belfast BT4 3SD, UK; Adrian.Allen@afbini.gov.uk (A.A.); Jordon.Graham@afbini.gov.uk (J.G.); Eleanor.Breadon@afbini.gov.uk (E.P.); Robin.Skuce@afbini.gov.uk (R.S.); 2 Veterinary Epidemiology Unit, Department of Agriculture, Environment and Rural Affairs, Belfast BT4 3SB, UK; Raymond.Kirke@daera-ni.gov.uk; 3 Department of Agriculture, Environment and Rural Affairs, Veterinary Service Animal Health, Coleraine BT52 2AJ, UK; Carl.McCormick@daera-ni.gov.uk; 4 School of Biological Sciences, Queen’s University Belfast, Belfast BT9 5DL, UK; AndrewW.Byrne@agriculture.gov.ie; 5 One-Health Unit, Department of Agriculture, Food and the Marine, Agriculture House, Dublin, Ireland

**Keywords:** bovine tuberculosis, molecular epidemiology, spatial, badgers, MLVA, Northern Ireland

## Abstract

Bovine tuberculosis surveillance in Northern Ireland includes Multiple-Locus Variable number tandem repeat Analysis (MLVA) to determine the *Mycobacterium bovis* genetic type present in both cattle and the predominant wildlife host, the European badger (*Meles meles*). These data are useful for investigating clusters of infection and understanding the scale at which interspecific transmission may occur. We utilised a comprehensive dataset of routinely sampled isolates from infected cattle and from badgers killed in road-traffic accidents to investigate the spatial co-location of MLVA types in, and between, the badger and cattle populations. Furthermore, we investigated the hypothesis that the type of farming enterprise might explain some variation in this relationship. MLVA types were spatially co-localised in cattle and road-traffic accident (RTA) badger hosts, indicative of a shared epidemic. Dairy herds were more likely to have at least one MLVA type in common with nearby RTA badgers, compared to non-dairy herd types. Marginally more MLVA spatial clustering was observed in non-dairy herds, which may be a consequence of relatively more between-herd movements. For the cattle population, local transmission mechanisms such as infection from contiguous herds, infectious wildlife and short-range between-herd cattle movements appear primarily to drive the epidemic: there appears to be a more limited role for long-range movements. Animal management practices are likely to be the driving force behind this observation, as beef rearing is associated with elevated numbers of animal movements compared to dairy herds.

## 1. Introduction

The wildlife-livestock interface presents a conduit through which pathogens can be exchanged [1]. In the UK and Ireland, the presence of wildlife reservoirs is implicated in the persistence of *Mycobacterium bovis*, the principal causative agent of bovine tuberculosis (bTB) in cattle [2,3]. *M. bovis* can infect a wide range of hosts, both wild and domesticated [4]. In the United Kingdom (UK) and the Republic of Ireland (ROI), the most important wildlife maintenance host is the European badger, *Meles meles* [5,6,7,8]. Infection transmission may be as a result of direct contact between species [9], or potentially through contaminated shared environments and fomites [10,11]. 

In Northern Ireland (NI), the costs of bTB control in cattle have exceeded £365 million over a recent twelve year period [12] and, despite an intensive and costly state-led programme focusing on the cattle population, eradication has not yet been achieved [13]. NI contains a relatively small area (approx. 13,500 km^2^), yet sustains a badger population of approximately 33,500 individuals (95% CI 26,000–41,200); [14]. A passive road-traffic accident (RTA) surveillance programme for *M. bovis* in badgers has been ongoing since 1998. This survey estimated *M. bovis* prevalence in sampled badgers to be 15.3% (95% CI 13.1–17.5%) [15,16], and revealed elevated bTB risk in cattle herds in close proximity to infected RTA badgers, compared to herds proximal to uninfected badgers [15]. Routine surveillance efforts for *M. bovis* in cattle and badger hosts also include spoligotyping and Multiple-Locus Variable number tandem repeat Analysis (MLVA) typing [17,18]. These data revealed clear spatial structuring (i.e., clustering) of *M. bovis* genetic types in cattle herds [18,19], and have shown that both cattle and badger hosts with the same MLVA type tend to be closer together than hosts with a different MLVA type [20,21]. This observed structure in the *M. bovis* population in NI indicates that the bTB epidemic is co-localised between both wild and domestic hosts, consistent with some degree of transmission between wild and domestic species [7]. Nevertheless, cattle to cattle transmission also contributes to the maintenance of infection at both the local and national scales, e.g., via within-herd amplification (regardless of source), contact with nearby infected herds, or between-herd cattle purchases involving infected animals [7,22,23,24].

However, the influence of cattle management and trade, as a risk factor, on the *M. bovis* population structure in both cattle and badgers has been given little attention to date. Beef and dairy production systems differ across a number of factors which may influence transmission. For example, we showed recently that beef herds in NI were generally associated with more between-herd cattle movements than dairy herds, and also experience elevated MLVA richness at the herd-level [25]. Different herd types have specific between-herd contact patterns potentially linked to different infection pathways [22], with beef fattening herds appearing more susceptible to infection introduced by bought-in cattle than dairy herds, and indeed, in NI, there is elevated risk of bTB infection associated with the purchase of beef animals [26]. These differences in herd management may subsequently manifest in different spatial relationships in the clustering of *M. bovis* in, and between, infected cattle and badger populations. We therefore aimed to analyse spatial relationships in the *M. bovis* MLVA types found both in RTA badgers and cattle herds in NI to ultimately gain insight into the spread of the epidemic, both within and between hosts.

## 2. Results

### 2.1. Summary Statistics

**Figure 1:**
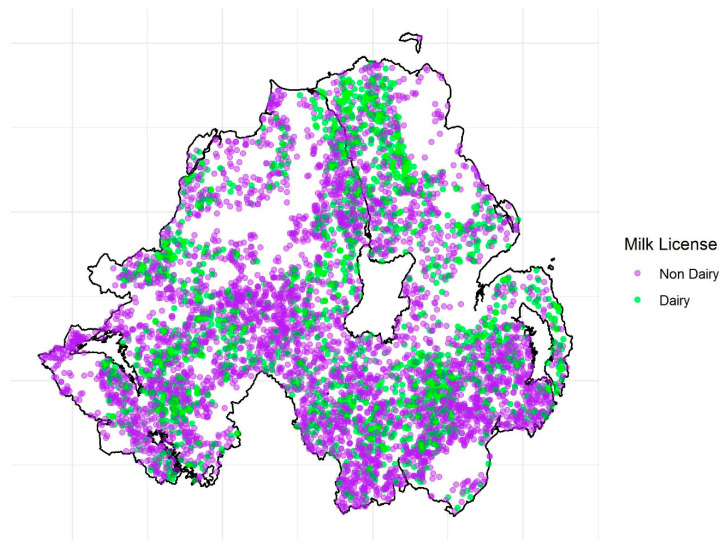
The spatial distribution of study herds with (green), and without (purple) a milk license in NI.

**Figure 2:**
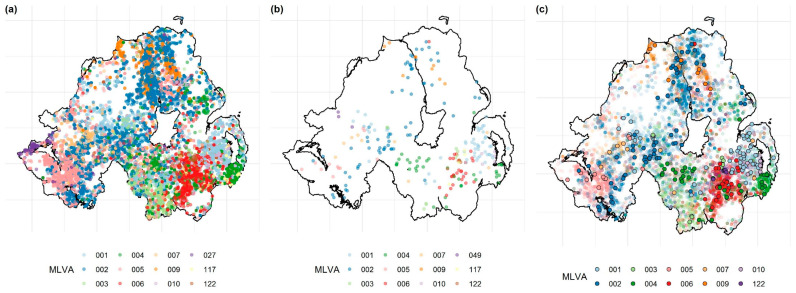
The spatial distribution of the twelve most common Multiple Locus Variable Analysis (MLVA) types in (**a**) cattle, (**b**) road-traffic accident (RTA) badgers, and (**c**) MLVA types common to both cattle and RTA badgers, where circles with a black outline represent isolates from RTA badgers, and circles without a black outline indicate cattle herds.

The final cattle dataset contained information on 9208 bTB breakdowns occurring between 2008 and 2016, in 6594 herds (herds with a milk license = 1822, 27.6%; without a milk license = 4772, 72.4%; see Figure 1). In total, 364 *M. bovis* MLVA types were isolated from the cattle population, 135 were found in both herds with and without milk licenses, 62 were found only in herds with milk licenses, and 167 were isolated only from herds without milk licenses. At herd level (i.e., the yearly herd-level incidence per MLVA type), the 12 most common MLVA types represented 77.8% of the total cattle isolates (Figure 2a; 001, 002, 003, 004, 005, 006, 007, 009, 010, 027, 117 and 122). The final RTA badger dataset contained data on 271 RTA badgers collected between 2008 and 2016, inclusive. Thirty different MLVA types were identified in this population, with the 12 most common MLVA types representing 90% of the total (Figure 2b). The spatial distribution of the most common herd-level *M. bovis* MLVA types present in both cattle and RTA badger hosts is shown in Figure 2c. In total, 26 (83.3%) of the 30 MLVA types found in badgers were also found in cattle. Herds with and without milk licenses differed across a number of epidemiologically relevant criteria, including herd size (herd size of herds with a milk license, median = 213; inter-quartile range (IQR): 123–338; without a milk license = 69; IQR: 33–137; wilcoxon signed rank test *p* < 0.001), outward movements (herds with a milk license = 23; IQR: 41–68; without a milk license = 21; IQR: 8–57; *p* < 0.001), and inwards movements (herds with a milk license = 1; IQR: 0–6; n without a milk license = 8; IQR: 0–43; *p* < 0.001).

### 2.2. Assessment of MLVA Clustering within the Cattle Population 

**Figure 3:**
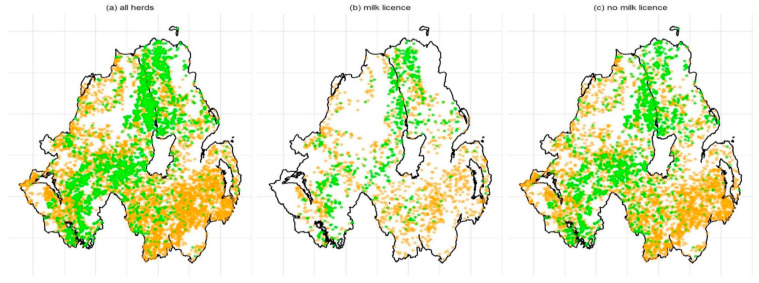
The spatial distribution of the most common MLVA type (002), shown in (**a**) all herds, (**b**) herds with a milk license, and (**c**) herds without a milk license. Green dots represent herds from which the MLVA type was isolated at least once, and orange dots represent herds from which the MLVA type was never isolated.

**Figure 4:**
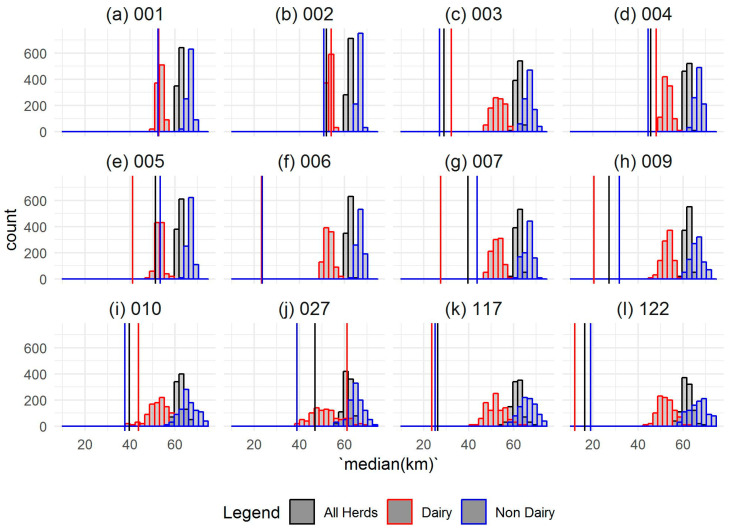
Visualization of the permutation analysis for all herds (black), herds with a milk license (red) and herds without a milk license (blue). The histograms show the sampling distribution of median distances between all herds infected with each of the twelve MLVA types (**a**–**l**), generated from 999 random permutations of the data. Vertical lines show the actual observed median distances between herds infected with each MLVA type. A vertical line intersecting with a histogram of the same colour indicated that the observed median distance between herds infected with the same MLVA type was not distinguishable from the median distances in a random selection of herds. The observed distances between all herds, dairy herds and non-dairy herds infected with types 001 and 006 were in very close agreement, and therefore appear as single lines on the figure.

Figure 3 illustrates the distribution of the most common MLVA type (002) in cattle in: (i) all cattle herds; Figure 3a, (ii) herds with milk licenses; Figure 3b, and (iii) herds without milk licenses; Figure 3c (See Supplementary Material 1, Figures S1–S11 for the remaining plots). The results of permutation analyses are visualized in Figure 4, which shows the actual median distance between herds infected with each MVLA type (vertical lines) compared to the distances between herds from the sampling distribution (histograms). For each MLVA type in all herds and non-dairy herds, the median distance between infected herds was significantly different from the distances in the sampling distribution (*p* < 0.05). In all cases, the actual, observed median distances were smaller than those from the sampling distribution, indicating that herds infected with the same MLVA type tended to be closer together than a random selection of herds. Similar results were obtained for herds with a milk license, with the exception of herds infected with types 001, 002, 010 and 027. In these cases, the actual median distance between infected herds did not differ significantly from the median distances obtained in the sampling distribution (*p* > 0.05). This can be observed for these MLVA types in Figure 4, where the values at the red vertical lines (distances between herds infected with each MLVA type) were not distinguishable from the red sampling distribution.

Table 1 shows the values (in km) of the 25th, 50th, 75th and 100th percentiles for between-herd pairwise distances for all herds, herds with a milk license, and herds without a milk license (see also See Supplementary Material 2, Figures S12–S23). The values for the 25th, 50th and 75th percentiles were furthermore reported as a percentage of the 100th percentile. The maximal distance between any two herds sharing the same MLVA type could be considerable, with observed distances of 167.3 km between infected herds (e.g., type 005, all herds). In all but one MLVA type (type 006), the overall maximal extent was smaller in herds with milk licenses than in herds without. Infection was considered spatially clustered if 50% of pairwise distances were found within 50% of the maximal pairwise distance. Indeed, the results show that, for all herds, 50% were found between 11.1% (type 022, all herds) and 35.5% (type 001, all herds) of the maximal pairwise distance between any two herds infected with the same MLVA type. This reflects a general trend of localised spatial clustering, with the majority of infected herds within relatively close proximity to each other, and a smaller number of infected herds disproportionally widely distributed. For only those herds with a milk license, 50% of herds were within 15.9% (type 009, dairy herds) to 44.8% (type 027, dairy herds) of the maximal pairwise distance between any two herds with a milk license infected with the same MLVA type. For eight MLVA types (001, 002, 003, 004, 122, 010, 027 and 117), marginally less localised clustering was observed in dairy herds compared to non-dairy herds.

### 2.3. Intra and Interspecific Nearest Neighbour (NN) Analysis

The median distance between NN RTA badgers which shared an MLVA genotype was 2.44 km (Inter-Quartile Range; IQR: 1.22–4.04 km), whilst the median distance between nearest neighbour RTA badgers which did not share an MLVA genotype was 3.33 km (IQR: 1.94–5.22 km; Wilcoxon signed rank test; V = 2853, *p* = 0.011). The distance between an RTA badger and the NN cattle herd in which the MLVA type was not isolated (1.49 km; IQR: 0.83–2.25 km) was almost 45% larger than the distance to a NN herd with the same MLVA type (0.82km; IQR: 0.50–1.64 km; V = 9469, *p* < 0.001). Similar findings were obtained for RTA badgers in proximity to both beef and dairy herds, see Table 2. The NN distance for cattle herds sharing an MLVA type was over 25% greater (1.12 km; IQR: 0.61–2.32 km) than the NN distance between herds which did not share the same MLVA type (0.82km; IQR: 0.51–1.27 km). This was also observed in non-dairy herds and, to a lesser extent, in dairy herds; see Table 2. All findings were also replicated in the sensitivity analysis (all *p* < 0.05), indicating that the spatial resolution of the data has no substantive impact on the interpretation of the results.

### 2.4. Distance-Based Similarlty Analysis

**Figure 5:**
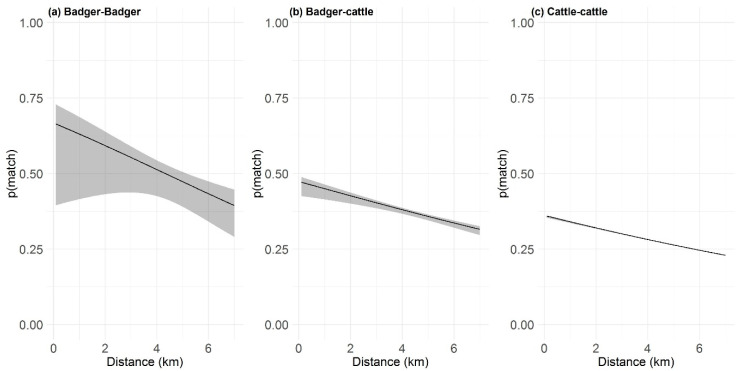
The relationship between probability of an MLVA match between two hosts as a function of distance in the (**a**) badger-badger context, (**b**) badger-cattle herds context, and (**c**), cattle-cattle herds context. Black lines show the fitted logistic regression, and the grey areas illustrate the 95% Confidence Intervals. The y-axis shows the probability that two hosts share a matching MLVA type; *p(match)*, as the distance between the hosts increases, shown on the x-axis.

Each RTA badger was surrounded by an average of four other badgers (IQR: 2–7) within a 7 km radius. The probability of two RTA badgers sharing the same MLVA type dropped by 17% for every km increase in distance between them (Odds Ratio; OR: 0.86; 95% lower and upper confidence limits; 95% CI: 0.80–0.92; Inverse OR: 1.17); Figure 5a. There were 63 cattle herds (IQR: 39–81) in the 7 km radius around each RTA badger, and the probability of RTA badgers and herds sharing MLVA types fell by 9% with every km (OR: 0.91; 95% CI: 0.90–0.92; Inverse OR: 1.09); Figure 5b. When stratified by herd type, we found that each RTA badger was surrounded by 17 dairy herds (IQR: 10–27) and 41 (IQR: 23–59) non-dairy herds, respectively. The probability of RTA badgers and dairy herds sharing MLVA types fell by 7% per km increase in distance (OR: 0.94; 95% CI: 0.92–0.95; Inverse OR: 1.07, and by 11% for non-dairy herds (OR: 0.90; 95% CI: 0.89–0.91; Inverse OR: 1.11). In the cattle-cattle context, each cattle herd was surrounded by 63 others (IQR: 40–88), and we found that the probability of two cattle herds sharing the same MLVA type dropped by 9% for every km increase in distance (OR: 0.91; 95% CI: 0.91-0.92; Inverse OR: 1.09); Figure 5c. There was a slight decrease when considering the dairy herd population independently (OR: 0.93; 95% CI: 0.93–0.94; Inverse OR: 1.07) from non-dairy herds (OR: 0.92; 95% CI: 0.91–0.92; Inverse OR: 1.09).

### 2.5. Determinng the Factors Associated with RTA Badgers and Nearby Cattle Herds Sharing MLVA Types

In the final dataset, there were 9471 RTA badger-cattle herd pairs with at least one MLVA type in common, and 5964 RTA badger-cattle herd pairs with no MLVA types in common. The univariable analysis revealed that the ‘number of MLVA types isolated from a bTB breakdown’ (OR per every additional type: 1.98; 95% CI: 1.83–2.13) and ‘herd size’ (OR per every additional ten animals: 1.01; 95% CI: 1.01–1.02) were positively associated with RTA badgers and nearby cattle herds sharing MLVA types. ‘Distance between hosts’ (OR per km: 0.89, 95% CI: 0.87–0.91), ‘the absence of a milk license’ (OR: 0.72, 95% CI: 0.67–0.77), ‘inwards movements’ (OR per every additional ten animals: 0.99; 95% CI: 0.99–0.99) and ‘outwards movements’ (OR per every additional ten animals: 0.98; 95% CI: 0.98–0.99) were negatively associated with RTA badgers and nearby cattle herds sharing MLVA types. The final model is presented in Table 3, and shows that the factors associated with RTA badgers and cattle sharing MLVA types were increasing ‘number of MLVA types during a breakdown’, ‘decreasing distance between hosts’, ‘the absence of a milk license’ and ‘fewer inwards movements’. No significant, biologically relevant interactions were identified. Herd size was correlated with the number of inwards movements (r = 0.63), was confounded with herd type and was therefore omitted from the final model; this did not impact Akaike information criterion (AIC) scores or model coefficients, notwithstanding that inwards movements was deemed to be the more relevant factor associated with the outcome.

## 3. Discussion

Spatial clustering in *M. bovis* molecular types, at various genetic and geographic scales, has previously identified co-localisation of infection between infected livestock and wildlife hosts [20,27,28,29,30,31]; however, little attention has been given to what the patterns in spatial distribution of *M. bovis* genetic types in NI reveal about the processes driving the epidemic in, and between, hosts. It is already understood that the *M. bovis* population in Northern Irish cattle herds is characterized by marked spatial structuring and spatial clusters of MVLA types at the herd level [18,19]. Here, we additionally show that the distribution of infection within clusters is not homogeneous, and that clusters consist of central foci, where 50% of infected herds lie within 35.5% of the cluster extent. This is consistent with “anchoring” influences in driving spatially restricted epidemics [32]; in Great Britain (GB), some 75% of infection was attributed to local spread [33]. Such processes act over relatively short distances, and can include infection from contiguous herds [26,34,35], infected wildlife [6,36,37], or the predominance of short-range, between-herd movements over longer-range movements, as observed in GB [38] and Ethiopia [39] (but not in Uruguay, where infection clusters change location year on year, suggesting long-distance spread of disease [40]). Spatial correlation has been reported in disease transmission coefficients at scales < 14 km, suggesting that a highly localised contact network is an important epidemiological driver of bTB [32]. However, we also identified pairwise distances of 167.3 km in herds infected with the same *M. bovis* MLVA type, and thus the spatial distribution of MLVA types also has an expansive element. Long-range cattle movements, or moving cattle between distal land-parcels, may drive the wider dissemination of infection [41,42], but our evidence is consistent with such processes being relatively less important than local factors investigated in driving the overall epidemic. This is in agreement with results from France, where spatial proximity to another infected herd was more strongly associated with bTB infection than inwards movements [43].

In our study, there was little compelling evidence of differences in the spatial dissemination of MLVA types between herds with and without a milk license, notwithstanding differences in inwards and outwards movements in both herd types. This observation may further reflect the diminished role of long-distance cattle movements in disease spread, compared to other sources. However, the data do tentatively indicate slightly less localised clustering in MLVA types in dairy herds, which could be explained by the fewer short-range, between-herd movements in dairy production. However, as yet the frequencies and Euclidian distances associated with the full cattle movement network in different herd types in NI are unknown [44]; whilst this study confirms that there are more inwards animal movements in non-dairy production, the distribution of trading distances between herds (as in Vernon, 2011 [38]) has not yet been derived. 

While there was greater richness in MLVA types in cattle herds compared to RTA badgers, the cattle population of 1.6 million is over 40 times larger than the badger population, estimated at 33,500 individuals (95% CI 26,000–41,200) [14], and may therefore be able to harbor a larger, more diverse microbial population [30]. Whilst the drivers of this are not yet clear, super-spreading, or historical expansions of the *M. bovis* population, may be implicated [45]. Indeed, this within-herd MLVA type diversity was revealed in the cattle-cattle nearest neighbor analysis, where it appeared that the neighboring herd which did not share an MLVA type was closer than the herd that did. This observation arose because more than one MLVA type can be present in cattle herds, and thus nearby herds can both share MLVA types, as well as exhibit differences. The nearest neighbor analysis was unable to account for this, whereas the dissimilarity analysis could better model the MLVA type diversity in the cattle population.

Over 80% of the *M. bovis* MLVA types identified in the RTA badger isolates were also found in cattle, and furthermore the infection was spatially co-localised in both hosts. This is consistent with previous findings using more limited herd-level data from NI [20], and data from GB and the Republic of Ireland (ROI) [27,28,29]. This association was clear, despite accepted limitations of both the badger and cattle data; farmstead locations are unlikely to represent actual land-parcel (or herd) locations, badgers killed in RTAs may not represent the background badger population, and the RTA dataset is spatially biased to the south-east of NI, and under-sampled in the north-west [16]. Given this, detecting associations despite these disruptive factors means that the actual spatial associations may be even stronger than observed. However, this would require a more thorough systematic sample of *M. bovis* infection in the extant NI badger population. While there is some work ongoing to collect such data [46], that study is limited to a small geographic area of NI (~100 km^2^). The probability of RTA badgers and cattle herds sharing *M. bovis* MLVA types decreased by approximately 9% for every km between hosts, up to a 7 km cut-off. Not only does this further confirm the co-localisation of infection in both host systems, but the relatively small rate of change also alludes to the strength of localised influenced in maintaining the *M. bovis* population structure, and by extension, the epidemic. There was greater similarity when looking at the likelihood of RTA badgers and herds with milk licenses sharing MLVA types (decrease of 7% per km) compared to RTA badgers and herds without milk licenses (decrease of 11% per km). Whilst this difference is small, it suggests greater between-herd homogeneity in the *M. bovis* population in herds with milk licenses, compared to herds without, possibly due to inwards movements driving accumulations of within-herd MLVA type diversity in non-dairy herds [25].

RTA badgers were more likely to have at least one MLVA type in common with herds with milk licenses than herds without. We posit that this reflects animal management practices, as beef herds are likely to operate by purchasing larger volumes of animals, retaining these animals in the herd for only a short period before sending cattle onwards or to slaughter. Beef herds have been linked to the presence of multiple reactors [26] arising from the purchase of animals with undetected infection, possibly from many different geographical locations. Indeed, we identified the inwards movement of animals as a negative influence on whether RTA badgers and cattle share MLVA types. We hypothesise that high cattle turnover in these herds means that the local MLVA types are less likely to become established in the immediate environment, limiting the opportunity for a co-localisation of *M. bovis* genotypes between badgers and cattle. The findings may also suggest that inter-species transmission is perhaps not particularly efficient [9] given that a shared *M. bovis* epidemic is less likely to be observed in herds associated with a high animal turnover. Conversely, animals in dairy settings may be more likely to be exposed to local *M. bovis* genotypes, resulting in the repeated emergence of *M. bovis* genotypes via introduction from local sources, possibly exacerbated by within-herd amplification. The diagnostic single intradermal comparative cervical tuberculin test (SICCT) may also be less effective in dairy herds [47] and infected dairy cattle may be less likely to exhibit visible lesions post mortem [48]. This is highly suggestive of the fact that dairy herds may be at elevated risk of within-herd recrudescence of the same *M. bovis* genotypes compared to beef herds.

### Limitations

As alluded to, the main limitation of this study is the nature of the RTA badger survey. The general limitations of these data are well acknowledged [16] but the bias in collection localities was presumed to have the most impact on these results. Nevertheless, this does not limit the utility of the dataset in making some inferences about the spatial relationships in MLVA types in, and between, the badger and cattle populations, with the caveat that a more representative RTA badger dataset would enable more robust inferences to be drawn. Ideally, a more comprehensive sampling of *M. bovis* genotypes in the badger population in NI would be undertaken which could permit the pathogen population structure to be derived in badger hosts. Furthermore, it is understood that cattle farms in NI are highly fragmented, and can consist of multiple, distal land parcels. The use of land parcel locations may provide a more accurate and precise indication of true herd locations and quantify the opportunity for herds to interact spatially with neighboring herds. This could shed more light on true spatial association of *M. bovis* genotypes in cattle herds. While co-localisation of molecular types between hosts, in various settings and at differing genetic and geographical scales, have been reported previously, our analyses extend our understanding of associated risk factors. Whilst molecular epidemiology using MLVA is being superseded by phylodynamics using whole-genome sequencing and modelling to investigate transmission dynamics [7], we argue for broad spatial scales, and for national monitoring there is still considerable value in using MLVA for bTB surveillance and epidemiological analysis.

## 4. Materials and Methods 

### 4.1. Study Area

The area of NI is approximately 13,500 km^2^. The bTB programme is administered across 10 Divisional Veterinary Offices (DVOs) and 123 administrative patches. There are approximately 1.6 million cattle in NI, distributed throughout approximately 20,000 herds. This includes some 2500 dairy herds (313,549 cattle) and 14,000 beef herds (247,009 cattle), amongst others [49]. 

### 4.2. Study Data

#### 4.2.1. *M. bovis* Molecular Typing Data

Whilst herd-level MLVA surveillance (MLVA typing on the first reactor) has been ongoing since 2003, from 2008, animal-level *M. bovis* MLVA typing is carried out on every SICCT reactor and lesioned animal identified at routine slaughter (LRS). MLVA analysis was carried out using established high resolution methods [18,19,50]. The eight M. bovis variable numbers of tandem repeats (VNTR) loci genotyped were MV2163B/QUB11B, MV4052/QUB26A, MV2461/ETRB, MV1955/Mtub21, MV1895/QUB1895, MV2165/ETRA, MV2163/QUB11A and MV3232/QUB3232. VNTR results were concatenated into a Multi-Locus VNTR Analysis (MLVA) string which constituted the molecular type of the isolate; this string was further simplified in a local laboratory nomenclature which reflected the previously assessed herd-level prevalence of MLVA types. 

#### 4.2.2. Cattle Data

Animal-level MLVA profiles of isolates were associated with anonymised breakdown-level data made available from the Animal and Plant Health Information System database (APHIS) [51], administered by the Department of Agriculture, Environment and Rural Affairs (DAERA). This enabled the determination of the number of *M. bovis* MLVA types present in each confirmed bTB breakdown; this dataset has been described in full elsewhere [25]. Additional relevant epidemiological variables included in these data were the breakdown start and end dates, presence of a milk license (dairy herds) or no milk license (non-dairy herds), herd size at the time of bTB breakdown, the number of inwards and outwards cattle movements in the year before breakdown, and the herd DVO. The geo-referencing of registered homestead locations (here referred to as herd locations) were available in the form of the first four digits of the six figure Irish grid reference, which provides reasonable estimations of cattle herd density and distribution. These data are available in an anonymised format in Supplementary Data 1. Actual herd numbers have been removed, and herd locations were “jittered” by between −999 m and +999 m to protect herds from being identified. 

#### 4.2.3. Badger Data

The RTA badger dataset has been described previously [16,20,52]. Briefly, from 1998, the carcasses of badgers suspected to have died from accidental causes (e.g., road traffic accidents, RTAs) were collected by a wildlife officer from the Department of Agriculture, Environment and Rural Affairs (DAERA). Badger carcasses were checked for the presence of visible lesions consistent with tuberculosis, and defined tissues and bodily fluid samples were also collected for bacterial culture. Culture-confirmed *M. bovis* underwent further MLVA analysis by the Agri-Food and Biosciences Institute (AFBI) to determine the bTB spoligotype and MLVA genotype of the isolate. The geo-referenced collection location for RTA badgers was recorded to within 100 m of the actual location. This study included only badgers collected after 2008 to align temporally with the cattle data. These data are available in an anonymised format in Supplementary Data 2. Badger collection localities were “jittered” by between −999 m and +999 m.

### 4.3. Assessment of MLVA Clustering within the Cattle Population

To visualise the extent of spatial clustering of MLVA types, the distribution of the twelve most common MLVA types were plotted in geographic space. The presence of spatial clustering in MLVA in cattle herds was confirmed by a permutation analysis. Firstly, the median Euclidean distance between herds infected with a given MLVA type was derived. Next, a random sample of herds was selected from the cattle herd population, with the sample size equal to the number of herds infected with the MLVA type of interest. The median Euclidean distance between the herds in this sample was then calculated. This process was repeated 999 times to generate a sampling distribution of distances. Finally, the actual median distance between herds infected with each MLVA type was compared to those derived from the sampling distribution using Wilcoxon signed rank tests.

Next, a pairwise Euclidean distance matrix between all herds infected with the same MVLA type was generated. The cumulative frequency of these distances was used to investigate the spatial spread of herds within clusters; if the majority of pairwise distances lay below the distance represented by the 50th percentile, this indicated spatial clustering. However, if the pairwise distances were broadly distributed (i.e., 50% of pairwise distances equal to or greater than the 50th percentile), this suggested instead that infected herds are widely distributed across space. This process was also conducted separately for herds with and without milk licenses.

### 4.4. Intra- and Interspecific Nearest Neighbour Analysis 

Following similar methods to Trewby (2016) [20] and Abernethy et al. (2011) [15], the Euclidian Nearest Neighbour (NN) distance was calculated between each RTA badger to the closest RTA badger with the same *M. bovis* MLVA type, and to the closest RTA badger with a different *M. bovis* MLVA type. Only badgers within 7 km of each other were included, as this represents the approximate 95 percentile of the dispersal movement kernel of badgers in the ROI [53]. Furthermore, the RTA collection dates had to fall within two years of each other; it is reasonable to assume that a two year window will adequately capture co-localisation of infection without introducing ambiguity from associating entities across longer temporal windows. Similar NN distance measures were calculated for RTA badger-cattle herd pairs, repeated separately for only those herds with a milk license, and those without a milk license. Again, only badger and cattle hosts within 7 km of each other were included, and the RTA collection date and herd breakdown period had to be within two years of each other. Finally, NN distances were calculated between cattle-cattle pairs, under the same criteria. The 7 km limit was considered acceptable for cattle-cattle associations in this instance, as we do not presently have information on the Euclidian distances associated with between-herd cattle movements. The null hypothesis was that distances between hosts which share *M. bovis* MLVA types were not significantly different to distances between hosts that do not. This was tested using paired Wilcoxon signed rank tests, and *p* was established at ≤0.05. To interrogate any limitations in the resolution of spatial data (i.e., the RTA badger and cattle herd co-ordinates were each subject to a 100 m error), a sensitivity analysis was conducted whereby the analyses were re-run 100 times, with values between 1m and 99 m added to, or subtracted from, each of the cattle and badger latitude and longitude co-ordinates.

### 4.5. Distance Based Similarlity Analysis

Using the approach established by Goodchild et al (2012) [27], the Odds Ratio (OR) of an *M. bovis* MLVA type match was calculated as a function of distance between hosts that yielded MLVA-typed *M. bovis*, up to a distance of 7 km. The two-year temporal association was again applied to this analysis. These data were used in the construction of logistic Generalised Linear Mixed Models (GLMMs), with a binary outcome indicating whether or not the *M. bovis* MLVA types matched. The single explanatory fixed-variable was distance, and herd DVO was allowed to vary with a random intercept. 

### 4.6. Determining the Factors Associated with RTA Badgers and Nearby Cattle Herds Sharing MLVA Types

We modelled factors associated with RTA badgers and nearby cattle sharing the same MLVA type. The outcome of interest was ‘whether any cow in a herd within a 7 km radius of an RTA badger shared the badger MLVA type’, was entered as a binary variable (1 = yes, 0 = no), and was modelled via a binomial GLMM. This analysis was again limited to breakdowns occurring within two years before or after an RTA collection date. Explanatory variables were: the presence or absence of a milk license, the distance between an RTA badger and registered cattle homestead, herd size, the number of inwards and outwards cattle movements, and the number of MLVA types isolated from a bTB breakdown. Initial univariable analysis involved visual assessments of each predictor, including Cleveland dot plots and boxplots, and fitting loess curves to assess linearity in the logit. Covariates were then assessed for co-linearity using both multi-panel scatterplots and correlation values; variables with a correlation coefficient greater than 0.5 or less than −0.5 were considered for removal, with the aim of retaining the most biologically relevant predictor(s). DVO was allowed to vary with a random intercept. The final model was arrived at via a backwards stepwise routine [54], and the impact of variable removal at each stage was assessed by comparing model AIC values, examining changes in model coefficients and assessing confounding. Where potential confounding was identified, it was investigated by running separate analyses on the suspected confounders. The influence of outliers and influential points was assessed by re-running models with potential influential points removed, and comparing the model coefficients. 

All data processing and analyses were carried out using Microsoft Excel and R version 3.4.4 (R Core Team 2014). The packages *rgdal* [55], *rgeos* [56] and *ggplot2* [57] were used to create maps and figures, models were built using *lme4* [58], and *dplyr* [59] was used for data handling.

## Figures and Tables

**Table 1:** The distances associated with the 25th, 50th, 75th and 100th percentiles from a frequency distribution of pairwise distances between herds infected with a given MLVA type. The values of the 25th, 50th and 75th percentiles are shown as a percentage of the maximum extent (100th percentile).

MLVA	Dataset	Total Infected Herds	25th Percentile (km)	50th Percentile (km)	75th Percentile (km)	100th Percentile (km)
001	All	1141	28.7 km (19.3%)	52.7 km (35.5%)	77.6 km (52.2%)	148.5 km
001	Dairy	347	29.2 km (19.2%)	52.8 km (34.7%)	76.4 km (50.1%)	152.4 km
001	Non Dairy	794	27.8 km (16.9%)	52.5 km (31.9%)	78.2 km (47.5%)	164.5 km
002	All	1885	30.9 km (18.5%)	52 km (31.3%)	76.5 km (46.0%)	166.4 km
002	Dairy	545	28.3 km (18.9%)	54.1 km (36.1%)	80.9 km (54.0%)	149.9 km
002	Non Dairy	1340	31.1 km (18.7%)	50.8 km (30.6%)	74.5 km (44.8%)	166.4 km
003	All	460	14.9 km (10.0%)	29 km (19.4%)	51.2 km (34.2%)	149.6 km
003	Dairy	118	17.4 km (13.6%)	32.2 km (25.1%)	51.5 km (40.1%)	128.4 km
003	Non Dairy	342	13.5 km (9.1%)	27 km (18.1%)	50.7 km (33.9%)	149.6 km
004	All	736	26.1 km (16.7%)	45.6 km (29.1%)	64.2 km (41.0%)	156.5 km
004	Dairy	204	27.6 km (19.7%)	48.1 km (34.3%)	68.3 km (48.7%)	140.5 km
004	Non Dairy	532	25.4 km (16.3%)	44.4 km (28.6%)	62.7 km (40.4%)	155.3 km
005	All	1001	23.8 km (14.3%)	51.3 km (30.7%)	79.3 km (47.4%)	167.3 km
005	Dairy	266	17.9 km (12.0%)	41.2 km (27.4%)	77.6 km (51.7%)	150.1 km
005	Non Dairy	735	26.1 km (15.6%)	53.4 km (31.9%)	79.8 km (47.7%)	167.3 km
006	All	810	13.9 km (9.5%)	23.6 km (16.1%)	40.9 km (27.9%)	146.4 km
006	Dairy	241	12.9 km (8.8%)	23.2 km (15.9%)	44.7 km (30.5%)	146.4 km
006	Non Dairy	569	14 km (10.3%)	23.5 km (17.3%)	39.6 km (29.2%)	135.8 km
007	All	388	19.8 km (13.8%)	39.6 km (27.6%)	65.6 km (45.6%)	143.8 km
007	Dairy	120	14.3 km (11.0%)	27.6 km (21.3%)	57 km (44.1%)	129.4 km
007	Non Dairy	268	22.7 km (15.8%)	43.7 km (30.4%)	67.9 km (47.2%)	143.8 km
009	All	355	17.1 km (10.5%)	27.1 km (16.7%)	44.9 km (27.7%)	162.0 km
009	Dairy	140	12 km (9.4%)	20.4 km (15.9%)	30.6 km (23.9%)	128.1 km
009	Non Dairy	215	19.3 km (11.9%)	31.7 km (19.5%)	52.5 km (32.4%)	162.0 km
122	All	157	8.5 km (5.8%)	16.3 km (11.1%)	32.1 km (21.7%)	147.5 km
122	Dairy	60	6.6 km (8.5%)	11.9 km (15.2%)	22.7 km (29.1%)	77.9 km
122	Non Dairy	97	10.4 km (7.1%)	19 km (12.9%)	36.7 km (24.9%)	147.5 km
010	All	148	14.2 km (10.2%)	39.7 km (28.6%)	59.6 km (42.9%)	138.9 km
010	Dairy	46	15 km (12.0%)	43.7 km (35%)	59.9 km (48.1%)	124.7 km
010	Non Dairy	102	12.9 km (9.7%)	37.7 km (28.3%)	59.3 km (44.4%)	133.5 km
027	All	157	18.7 km (11.4%)	46.9 km (28.6%)	88.5 km (54.1%)	163.8 km
027	Dairy	26	27.8 km (20.3%)	61.2 km (44.8%)	84.1 km (61.6%)	136.4 km
027	Non Dairy	131	13.8 km (9.2%)	38.9 km (25.9%)	84.1 km (56.1%)	150.0 km
117	All	116	15 km (10.0%)	26.2 km (17.5%)	38.8 km (25.9%)	149.7 km
117	Dairy	37	12.3 km (9.0%)	23.7 km (17.3%)	37.4 km (27.3%)	136.8 km
117	Non Dairy	79	14.6 km (9.8%)	25.2 km (17.0%)	39.5 km (26.6%)	148.5 km

**Table 2:** Nearest-neighbour (NN) distances for pairs of RTA badgers and cattle herds which share MLVA types, and which do not share MLVA types.

	NN Distance for Hosts Which Share an MLVA Type			NN Distance for Hosts Which Do Not Share an MLVA Type			
	Median (km)	Q1 (km)	Q4 (km)	Median (km)	Q1 (km)	Q4 (km)	Difference in Medians (km)
Badger-Badger	2.44	1.22	4.04	3.33	1.94	5.22	0.89
Badger-Cattle (all herds)	0.82	0.50	1.64	1.49	0.83	2.25	0.67
Badger-Cattle (non-dairy)	1.14	0.61	1.94	2.55	1.62	3.74	1.41
Badger-Cattle (dairy)	1.70	0.85	2.73	2.88	1.85	4.03	1.18
Cattle-Cattle (all herds)	1.12	0.61	2.32	0.82	0.51	1.27	0.29
Cattle-Cattle (dairy)	1.60	0.85	2.95	1.39	0.82	2.02	0.21
Cattle-Cattle (non-dairy)	1.28	0.67	2.66	0.93	0.58	1.50	0.35

**Table 3:** Model coefficients for the factors associated with RTA badgers sharing at least one MLVA in common with nearby cattle herds. The median (Med) and Inter-Quartile Range (IQR) are reported for continuous variables, and where appropriate the maximum (Max) value is also included. The number of instances and percentages are reported for binary variables.

Variable	Match n = 9471 (61.4%)	No match n = 5964 (38.6%)	Est.	Std. Error	z Value	OR	95% CI Lower	95% CI Upper
Intercept	-	-	0.19	0.19	0.99	1.21	0.83	1.75
Number of MLVA types (per type)	Med: 1 IQR: 1–1; Max: 10	Med: 1; IQR: 1–1; Max: 5	0.85	0.04	20.28	2.34	2.16	2.54
Distance (per km)	Med: 4.4 km IQR: 2.9–5.7 km	Med: 4.9 km IQR: 3.3–6 km	−0.12	0.01	−11.61	0.89	0.87	0.91
Milk license (absent)	6083 (64.2%)	4261 (71.5%)	−0.28	0.04	−7.42	0.75	0.70	0.81
Inwards cattle movements (per 10 animals)	Med: 4 IQR: 2–9	Med: 5 IQR: 2–11	−0.02	0.001	−11.00	0.99	0.98	0.99

## Supplementary Materials

The following are available online at https://www.mdpi.com/2076-0817/9/7/592/s1; Supplementary Data 1 and Supplementary Data 2; Supplementary Material 1, Figure S1. The spatial distribution of MLVA type 001, shown in (a) all herds, (b) herds with a milk license, and (c) herds without a milk license. Green dots represent herds from which the MLVA type was isolated at least once; Figure S2. The spatial distribution of MLVA type 003, shown in (a) all herds, (b) herds with a milk license, and (c) herds without a milk license. Green dots represent herds from which the MLVA type was isolated at least once; Figure S3. The spatial distribution of MLVA type 004, shown in (a) all herds, (b) herds with a milk license, and (c) herds without a milk license. Green dots represent herds from which the MLVA type was isolated at least once; Figure S4. The spatial distribution of MLVA type 005, shown in (a) all herds, (b) herds with a milk license, and (c) herds without a milk license. Green dots represent herds from which the MLVA type was isolated at least once; Figure S5. The spatial distribution of MLVA type 006, shown in (a) all herds, (b) herds with a milk license, and (c) herds without a milk license. Green dots represent herds from which the MLVA type was isolated at least once; Figure S6. The spatial distribution of MLVA type 007, shown in (a) all herds, (b) herds with a milk license, and (c) herds without a milk license. Green dots represent herds from which the MLVA type was isolated at least once; Figure S7. The spatial distribution of MLVA type 009, shown in (a) all herds, (b) herds with a milk license, and (c) herds without a milk license. Green dots represent herds from which the MLVA type was isolated at least once; Figure S8. The spatial distribution of MLVA type 010, shown in (a) all herds, (b) herds with a milk license, and (c) herds without a milk license. Green dots represent herds from which the MLVA type was isolated at least once; Figure S9. The spatial distribution of MLVA type 027, shown in (a) all herds, (b) herds with a milk license, and (c) herds without a milk license. Green dots represent herds from which the MLVA type was isolated at least once; Figure S10. The spatial distribution of MLVA type 117, shown in (a) all herds, (b) herds with a milk license, and (c) herds without a milk license. Green dots represent herds from which the MLVA type was isolated at least once; Figure S11. The spatial distribution of MLVA type 122, shown in (a) all herds, (b) herds with a milk license, and (c) herds without a milk license. Green dots represent herds from which the MLVA type was isolated at least once. Supplementary Material 2: Figure S12. The Cumulative Density Function (CDF) of MLVA type 001. Figure S13. The Cumulative Density Function (CDF) of MLVA type 002. Figure S14. The Cumulative Density Function (CDF) of MLVA type 003. Figure S15. The Cumulative Density Function (CDF) of MLVA type 004. Figure S16. The Cumulative Density Function (CDF) of MLVA type 005. Figure S17. The Cumulative Density Function (CDF) of MLVA type 006. Figure S18. The Cumulative Density Function (CDF) of MLVA type 007. Figure S19. The Cumulative Density Function (CDF) of MLVA type 009. Figure S20. The Cumulative Density Function (CDF) of MLVA type 010. Figure S21. The Cumulative Density Function (CDF) of MLVA type 027. Figure S22. The Cumulative Density Function (CDF) of MLVA type 117. Figure S23. The Cumulative Density Function (CDF) of MLVA type 122.
