# Supplementary material for: Mycobacterium bovis Population Structure in Cattle and Local Badgers: Co-Localisation and Variation by Farm Type
Source: Pathogens. 2020 Jul 21;9(7):592. doi: 10.3390/pathogens9070592 (PMC7400278; doi:10.3390/pathogens9070592)
Supplement: Supplementary file 1 [file pathogens-09-00592-s001.zip › Supplementary Material 1.docx]

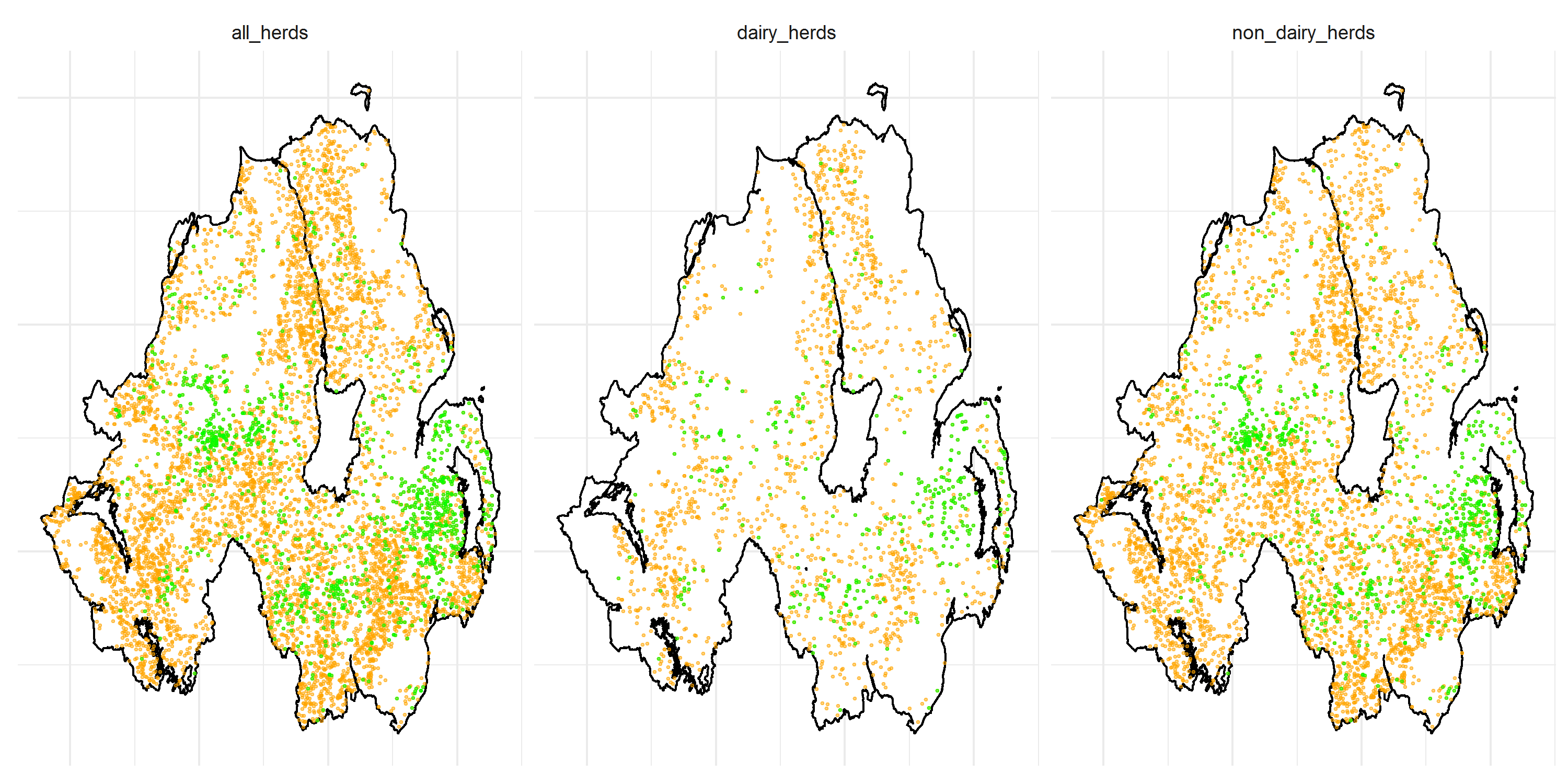


**Figure S1.** The spatial distribution of MLVA type 001, shown in (a) all herds, (b) herds with a milk license, and (c) herds without a milk license. Green dots represent herds from which the MLVA type was isolated at least once.


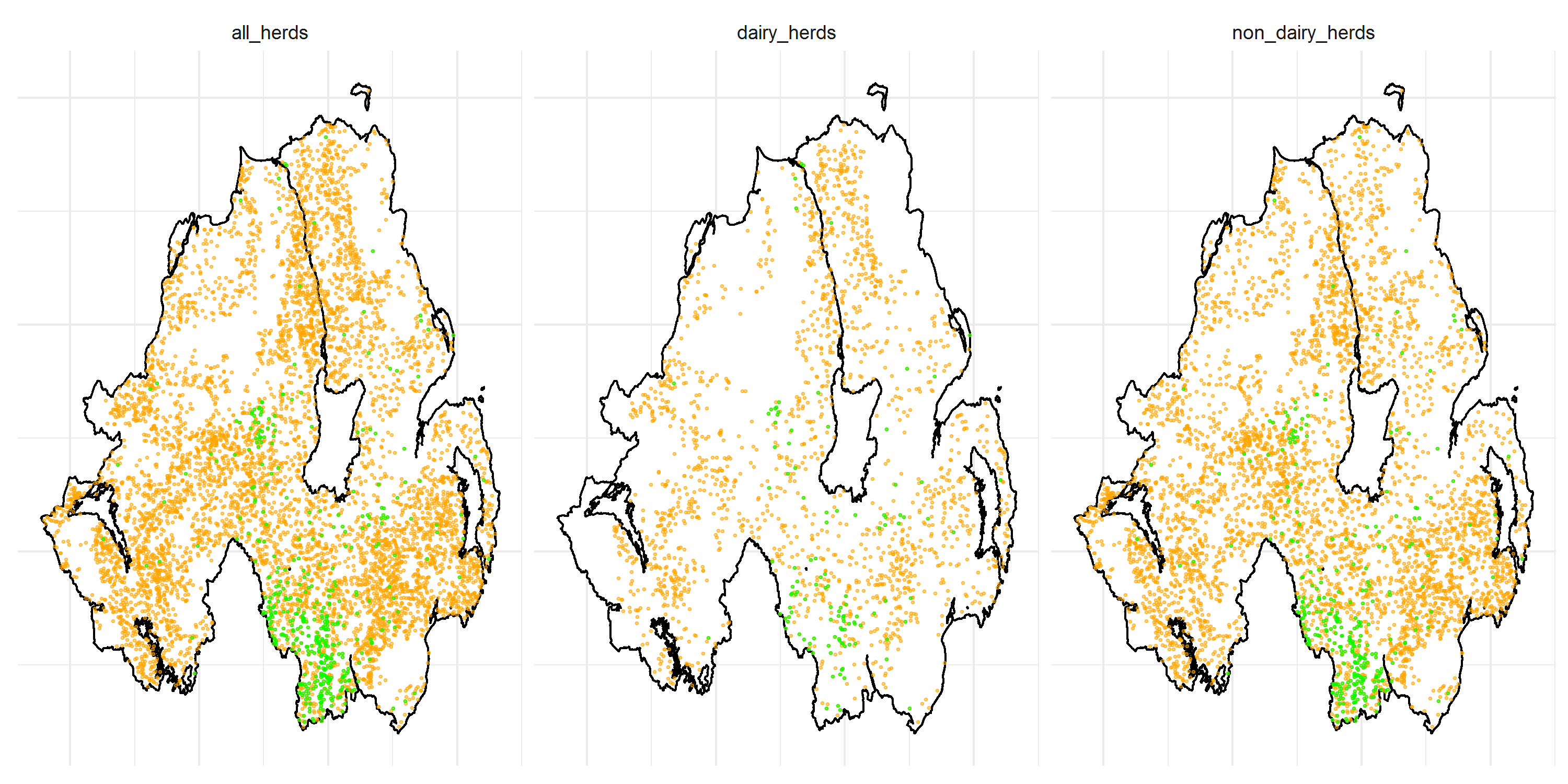


**Figure S2.** The spatial distribution of MLVA type 003, shown in (a) all herds, (b) herds with a milk license, and (c) herds without a milk license. Green dots represent herds from which the MLVA type was isolated at least once.

**
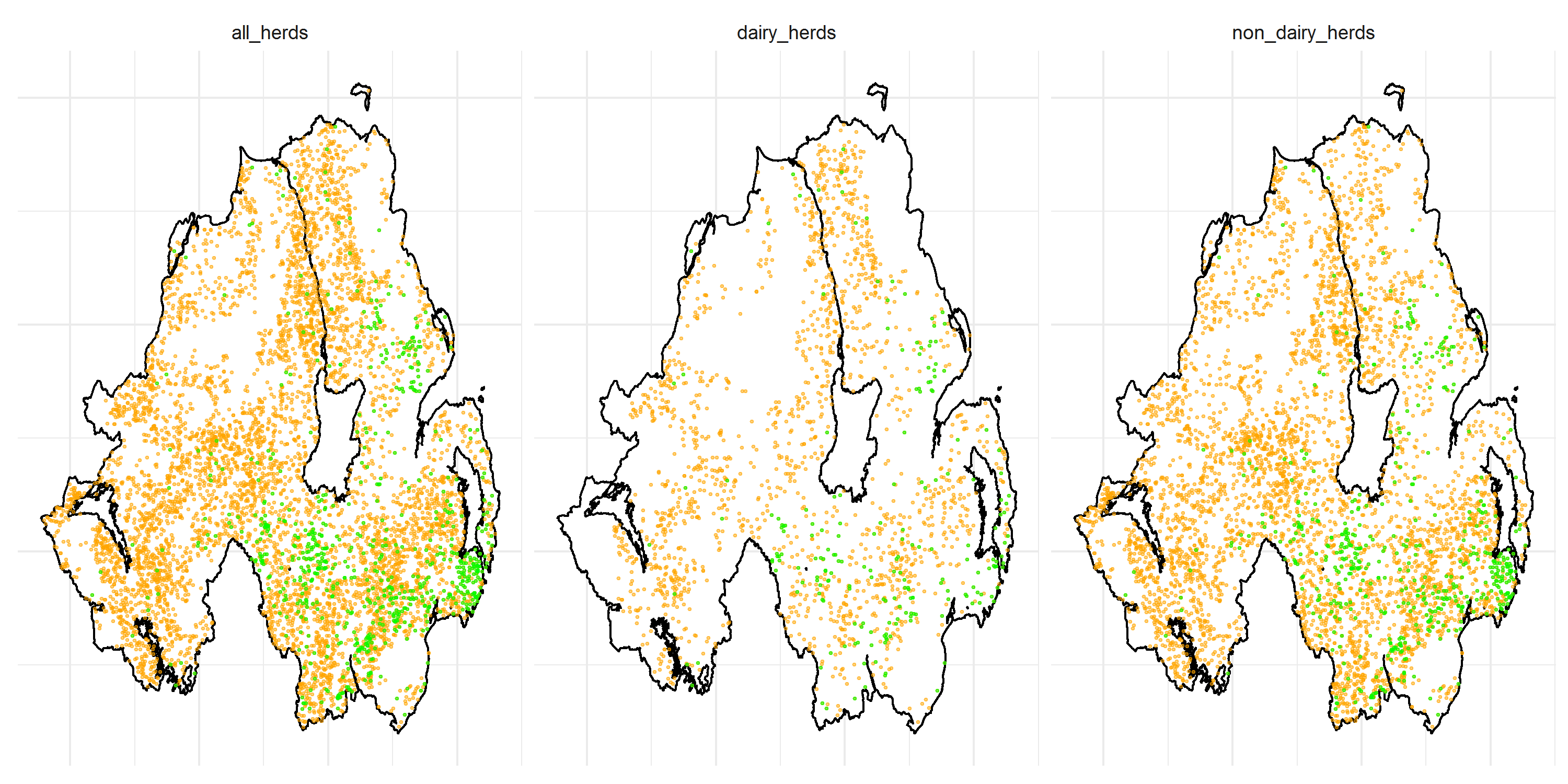
**

**Figure S3.** The spatial distribution of MLVA type 004, shown in (a) all herds, (b) herds with a milk license, and (c) herds without a milk license. Green dots represent herds from which the MLVA type was isolated at least once

**
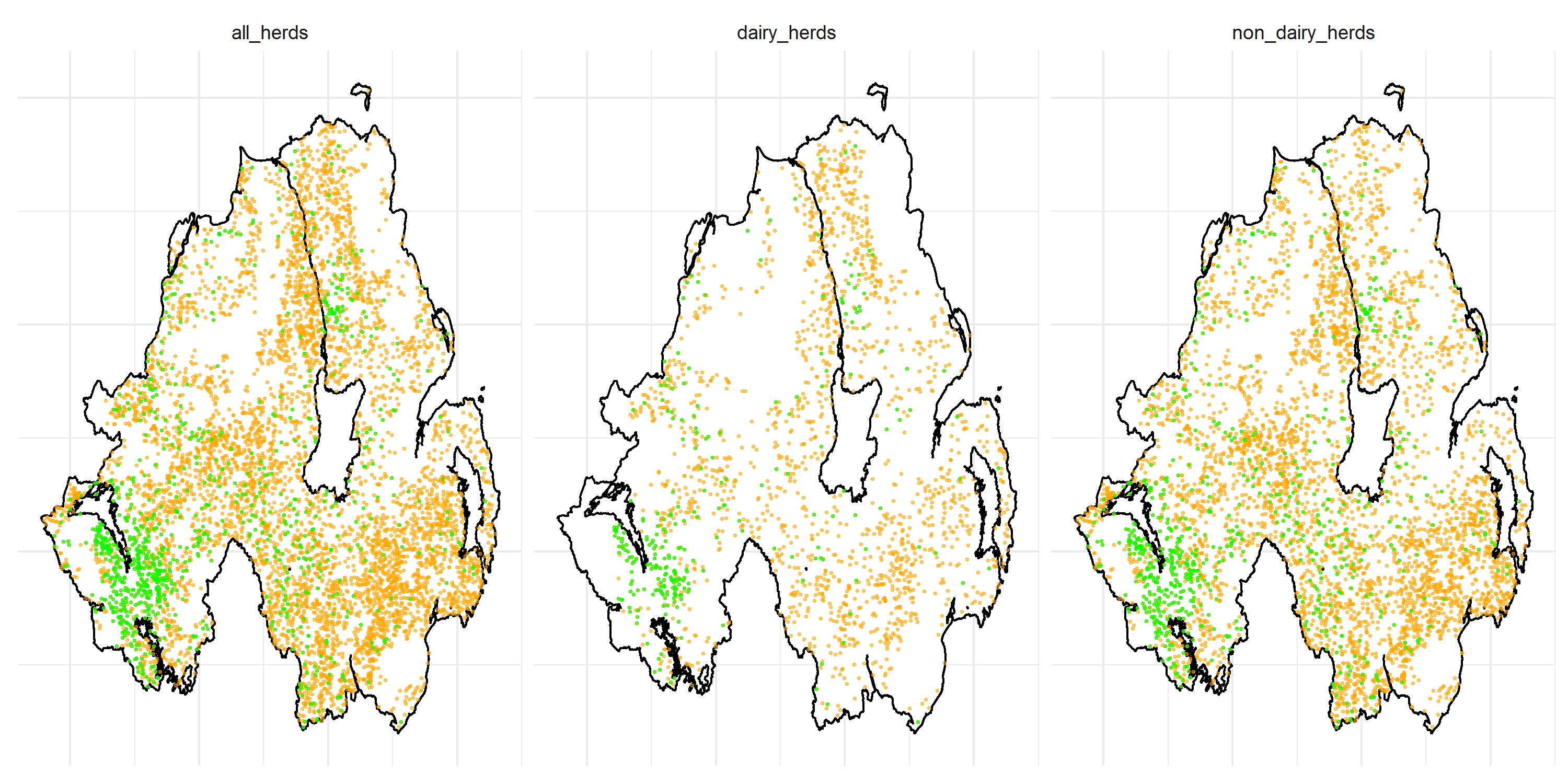
**

**Figure S4.** The spatial distribution of MLVA type 005, shown in (a) all herds, (b) herds with a milk license, and (c) herds without a milk license. Green dots represent herds from which the MLVA type was isolated at least once


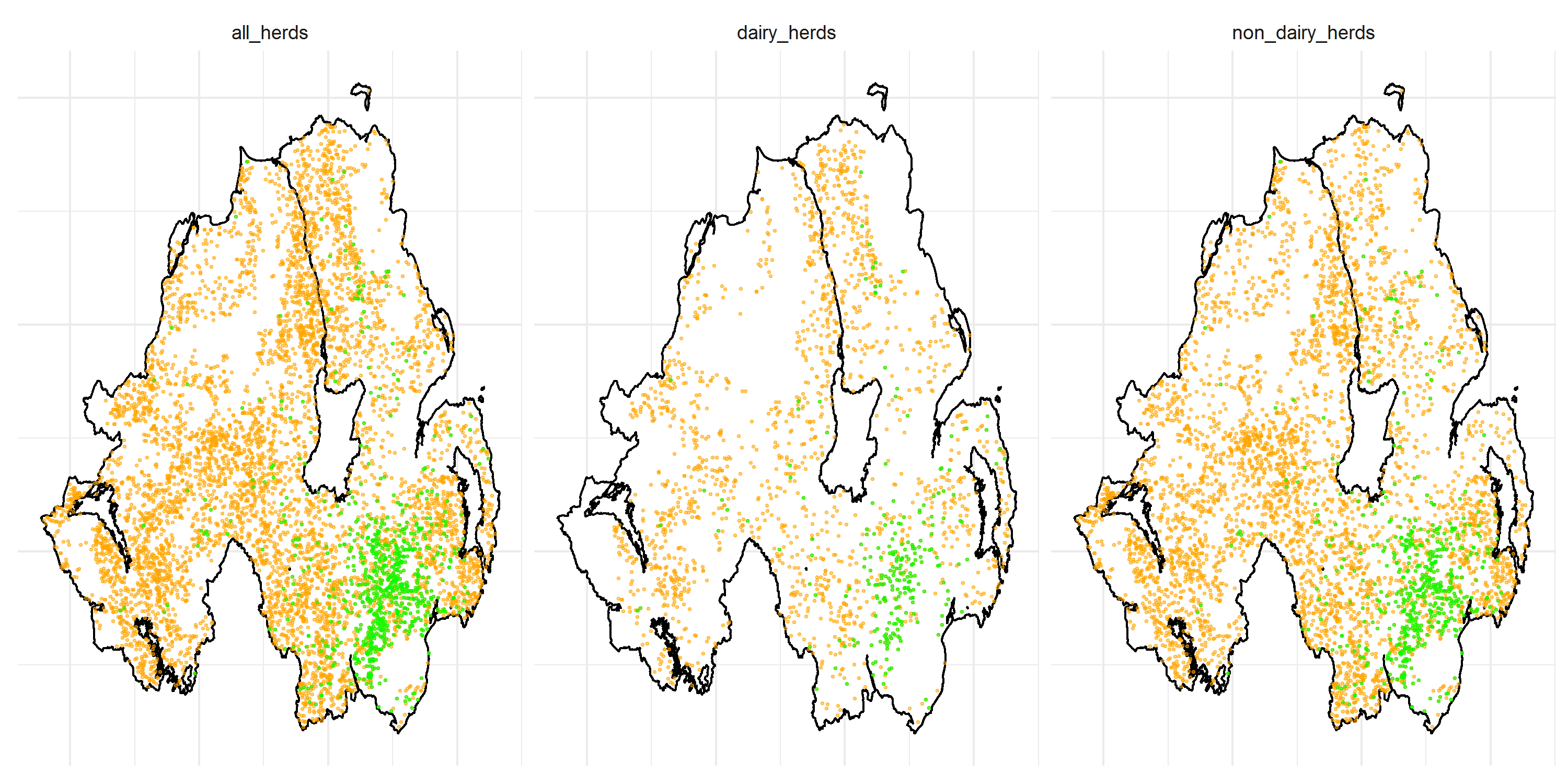


**Figure S5.** The spatial distribution of MLVA type 006, shown in (a) all herds, (b) herds with a milk license, and (c) herds without a milk license. Green dots represent herds from which the MLVA type was isolated at least once


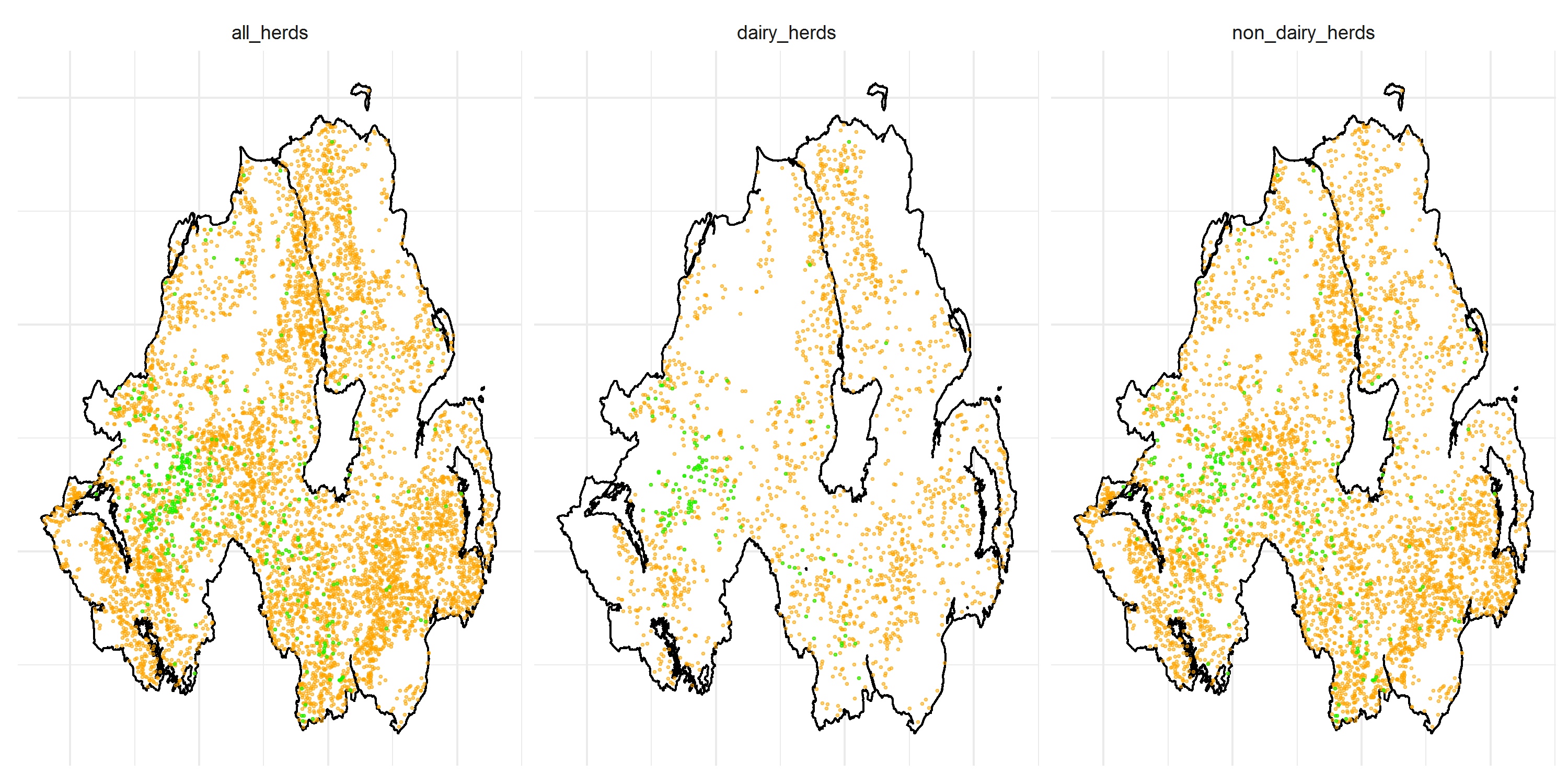


**Figure S6.** The spatial distribution of MLVA type 007, shown in (a) all herds, (b) herds with a milk license, and (c) herds without a milk license. Green dots represent herds from which the MLVA type was isolated at least once

**
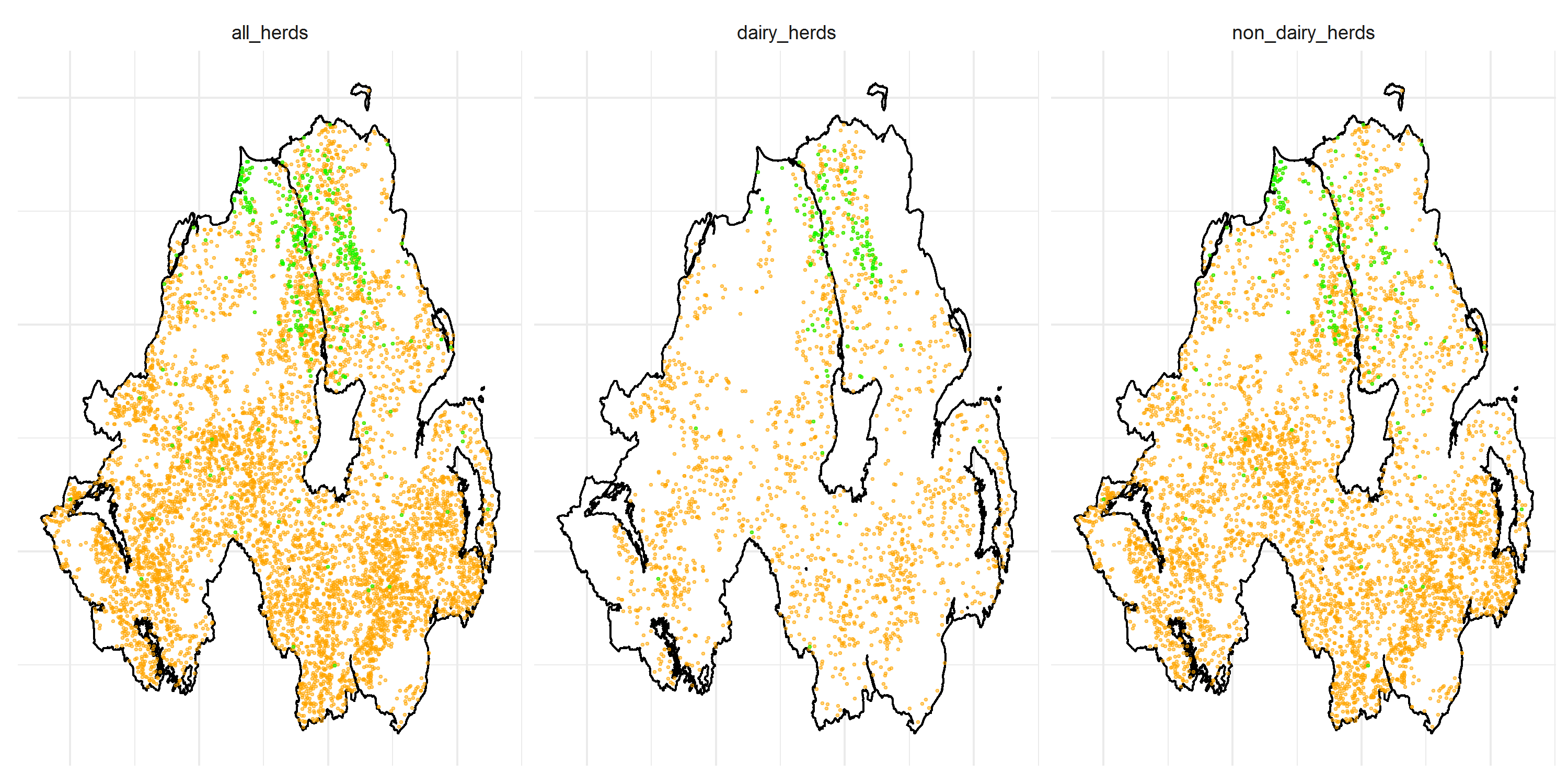
**

**Figure S7.** The spatial distribution of MLVA type 009, shown in (a) all herds, (b) herds with a milk license, and (c) herds without a milk license. Green dots represent herds from which the MLVA type was isolated at least once


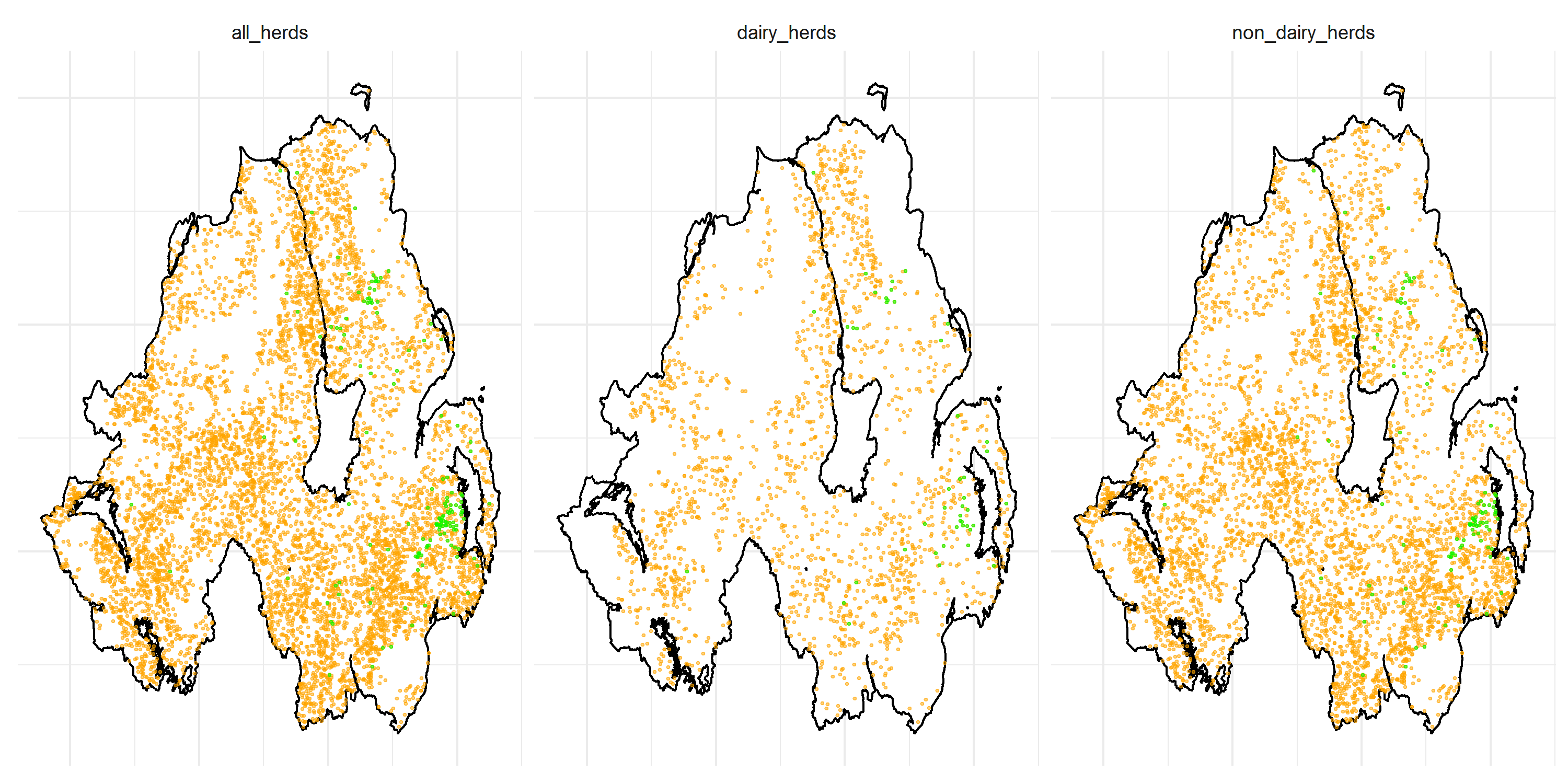


**Figure S8.** The spatial distribution of MLVA type 010, shown in (a) all herds, (b) herds with a milk license, and (c) herds without a milk license. Green dots represent herds from which the MLVA type was isolated at least once


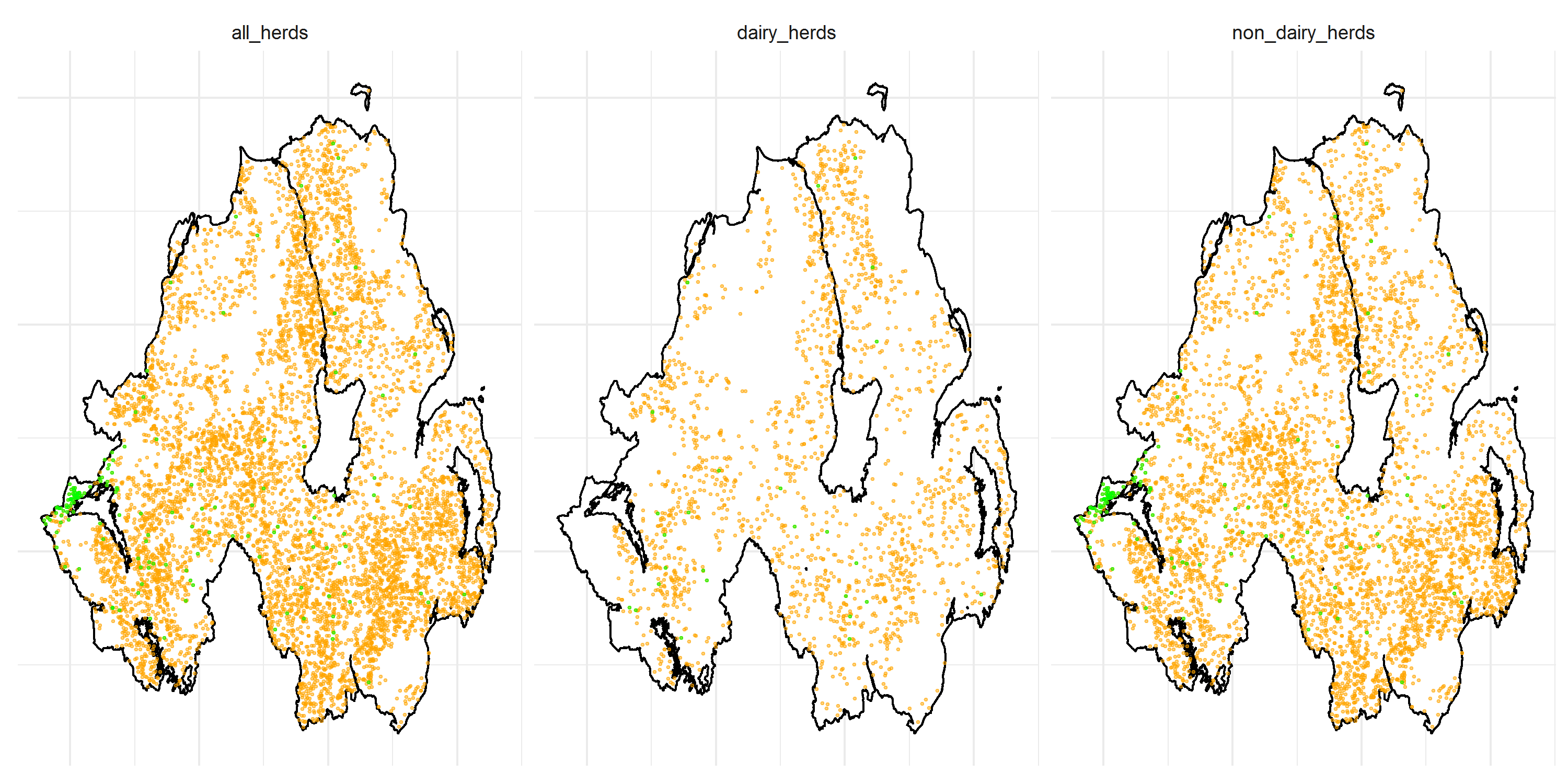


**Figure S9.** The spatial distribution of MLVA type 027, shown in (a) all herds, (b) herds with a milk license, and (c) herds without a milk license. Green dots represent herds from which the MLVA type was isolated at least once

**
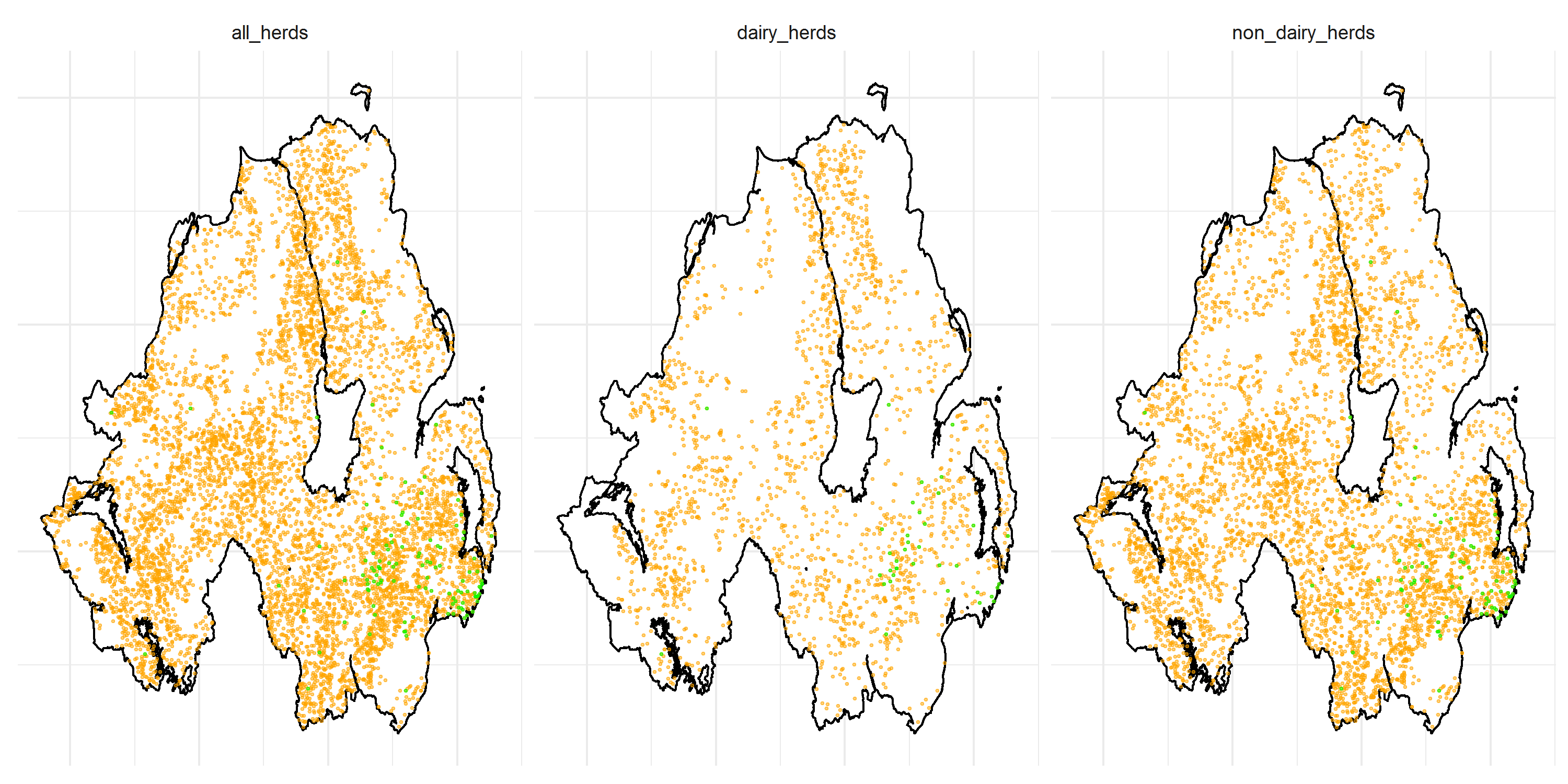
**

**Figure S10.** The spatial distribution of MLVA type 117, shown in (a) all herds, (b) herds with a milk license, and (c) herds without a milk license. Green dots represent herds from which the MLVA type was isolated at least once

**
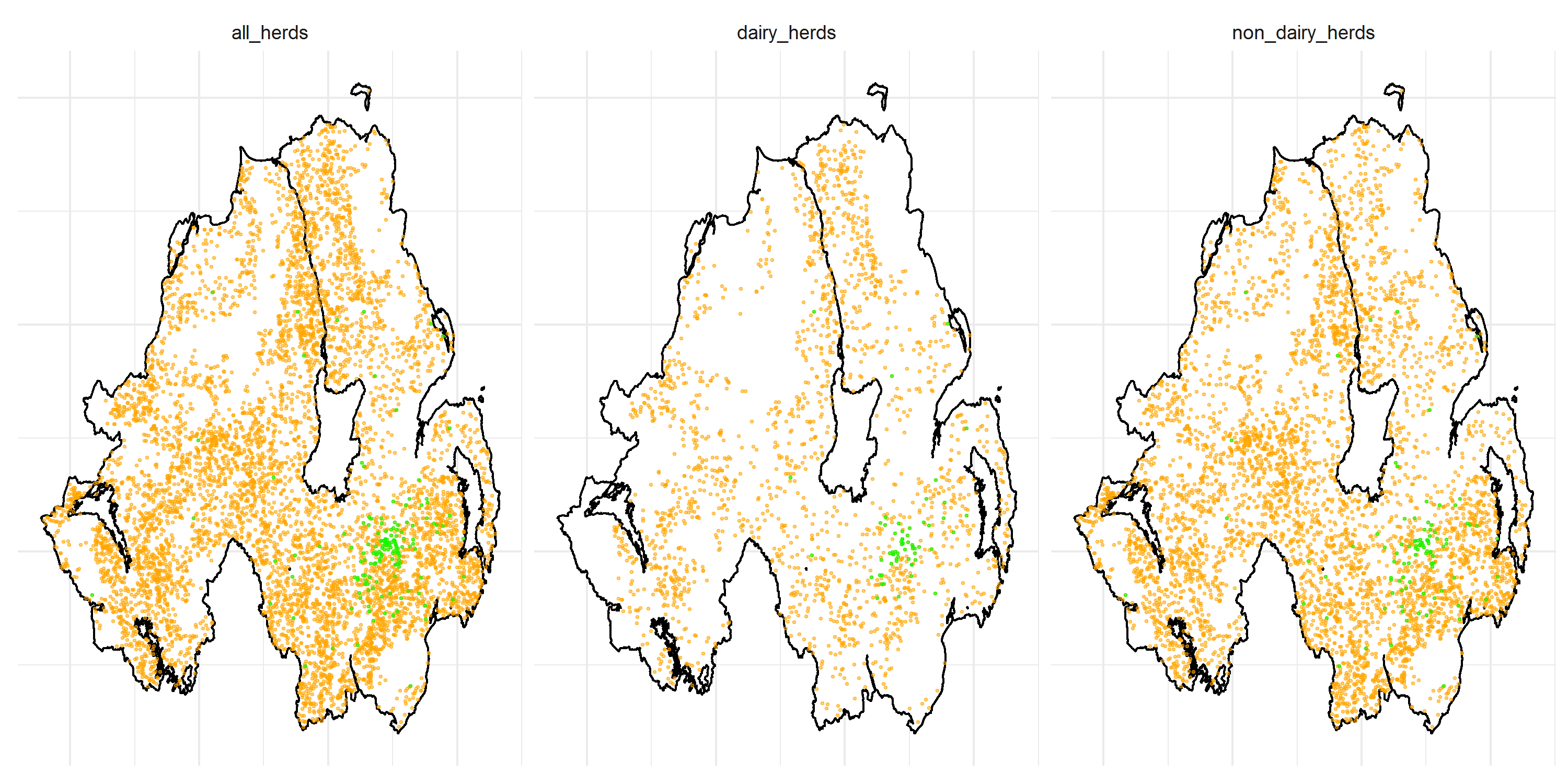
**

**Figure S11.** The spatial distribution of MLVA type 122, shown in (a) all herds, (b) herds with a milk license, and (c) herds without a milk license. Green dots represent herds from which the MLVA type was isolated at least once
