# Supplementary material for: Mycobacterium bovis Population Structure in Cattle and Local Badgers: Co-Localisation and Variation by Farm Type
Source: Pathogens. 2020 Jul 21;9(7):592. doi: 10.3390/pathogens9070592 (PMC7400278; doi:10.3390/pathogens9070592)
Supplement: Supplementary file 1 [file pathogens-09-00592-s001.zip › Supplementary Material 2.docx]

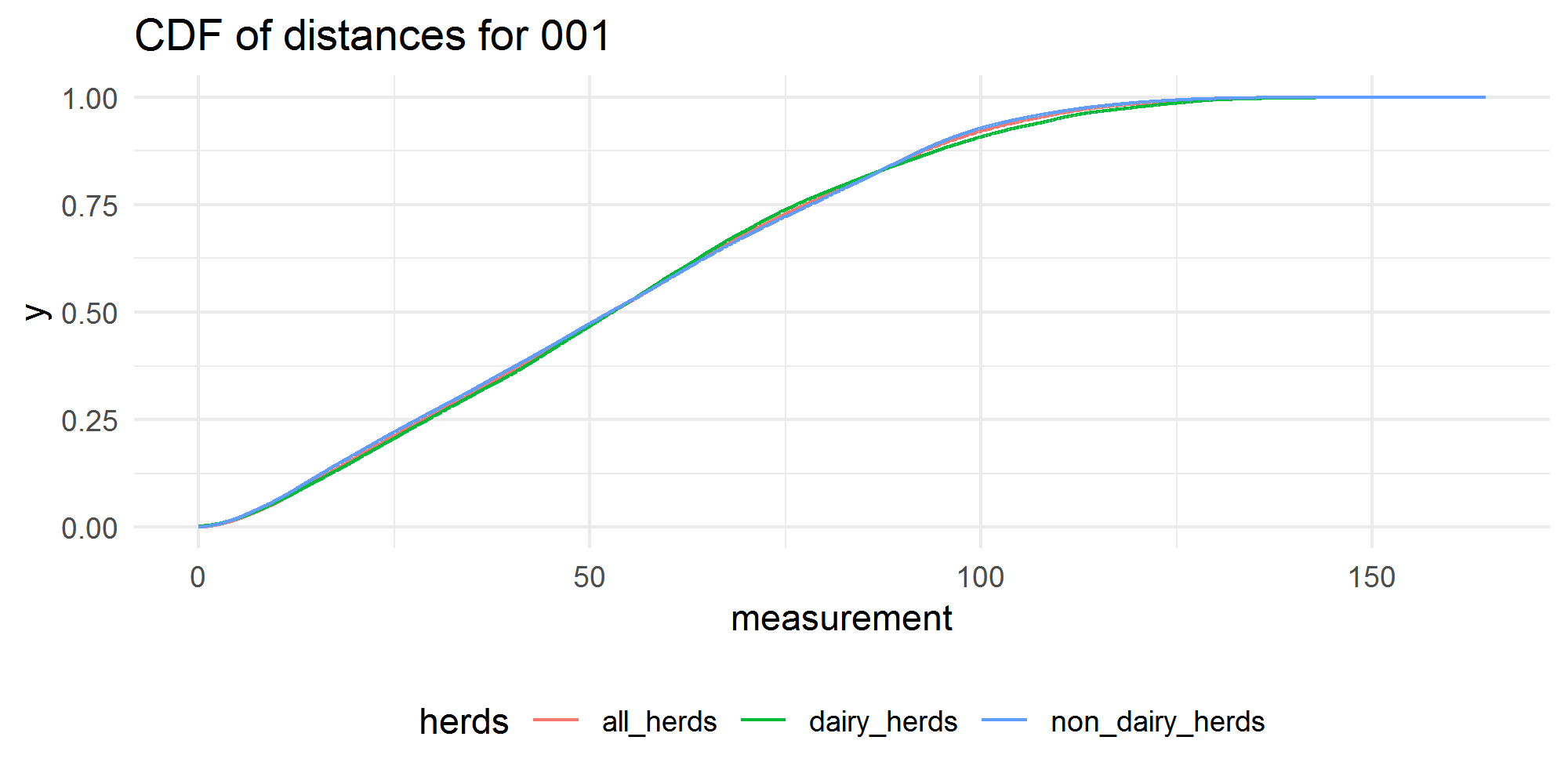


**Figure S12.** The Cumulative Density Function (CDF) of MLVA type 001.


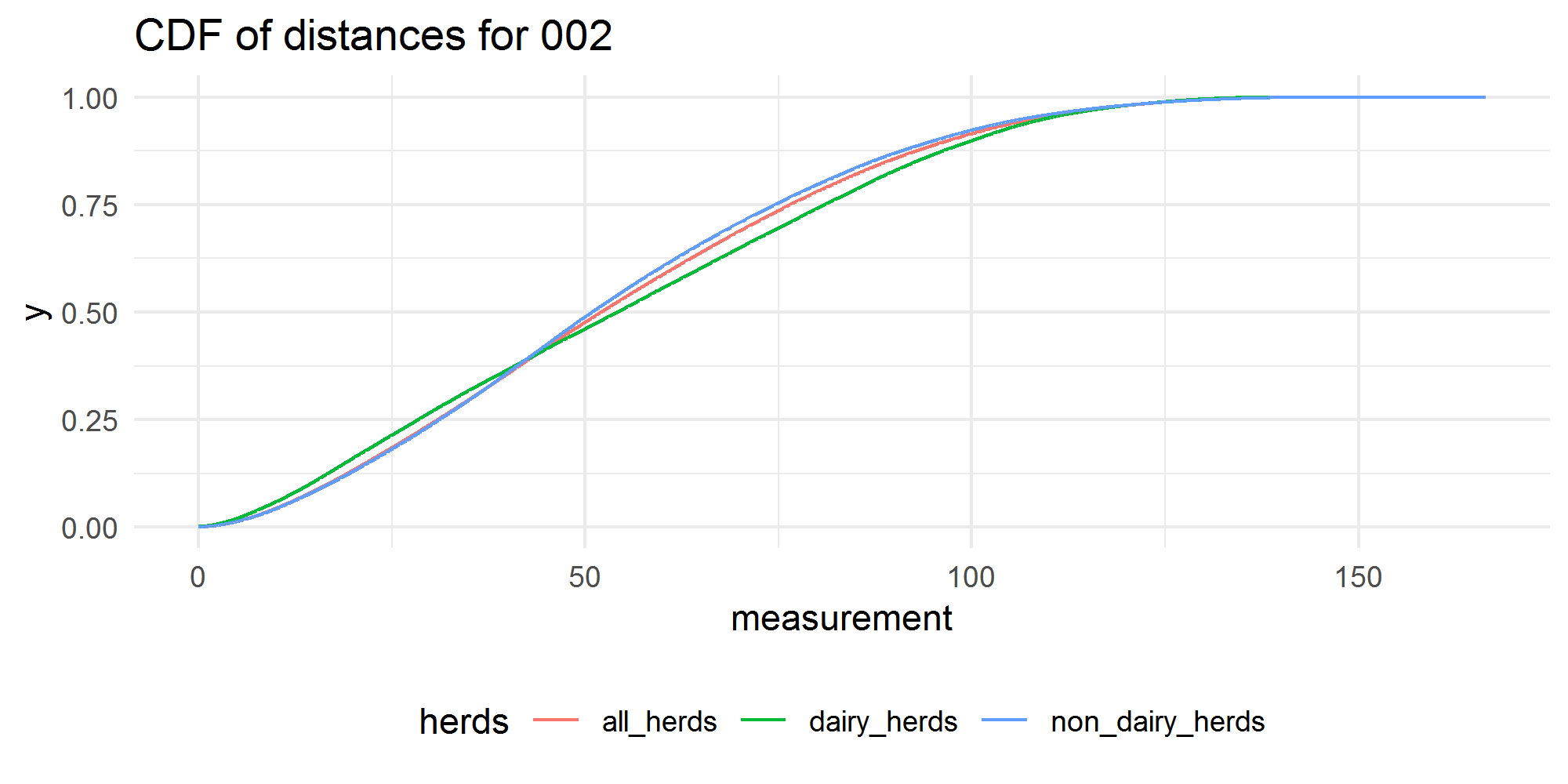


**Figure S13.** The Cumulative Density Function (CDF) of MLVA type 002.


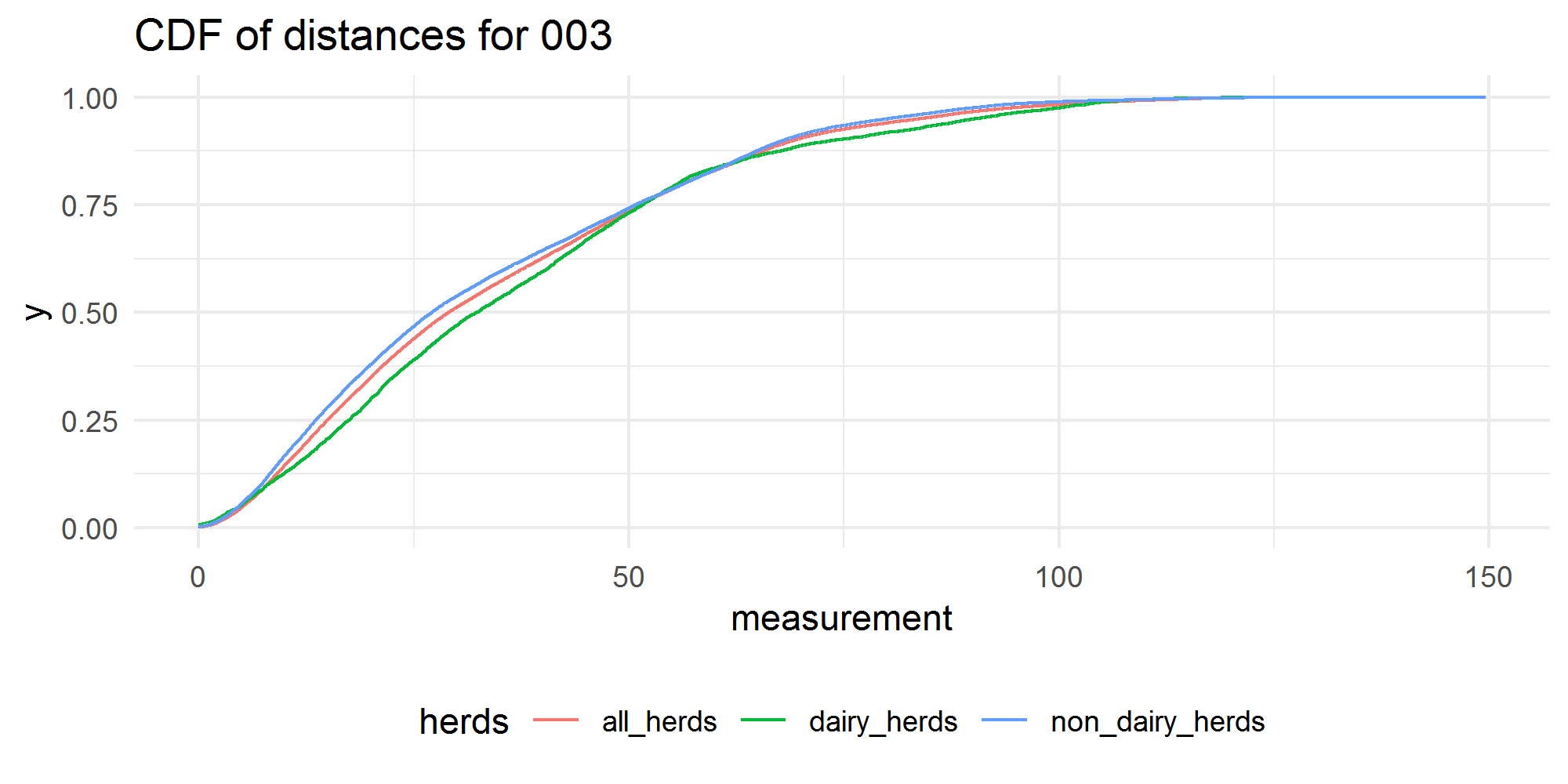


**Figure S14.** The Cumulative Density Function (CDF) of MLVA type 003.

**
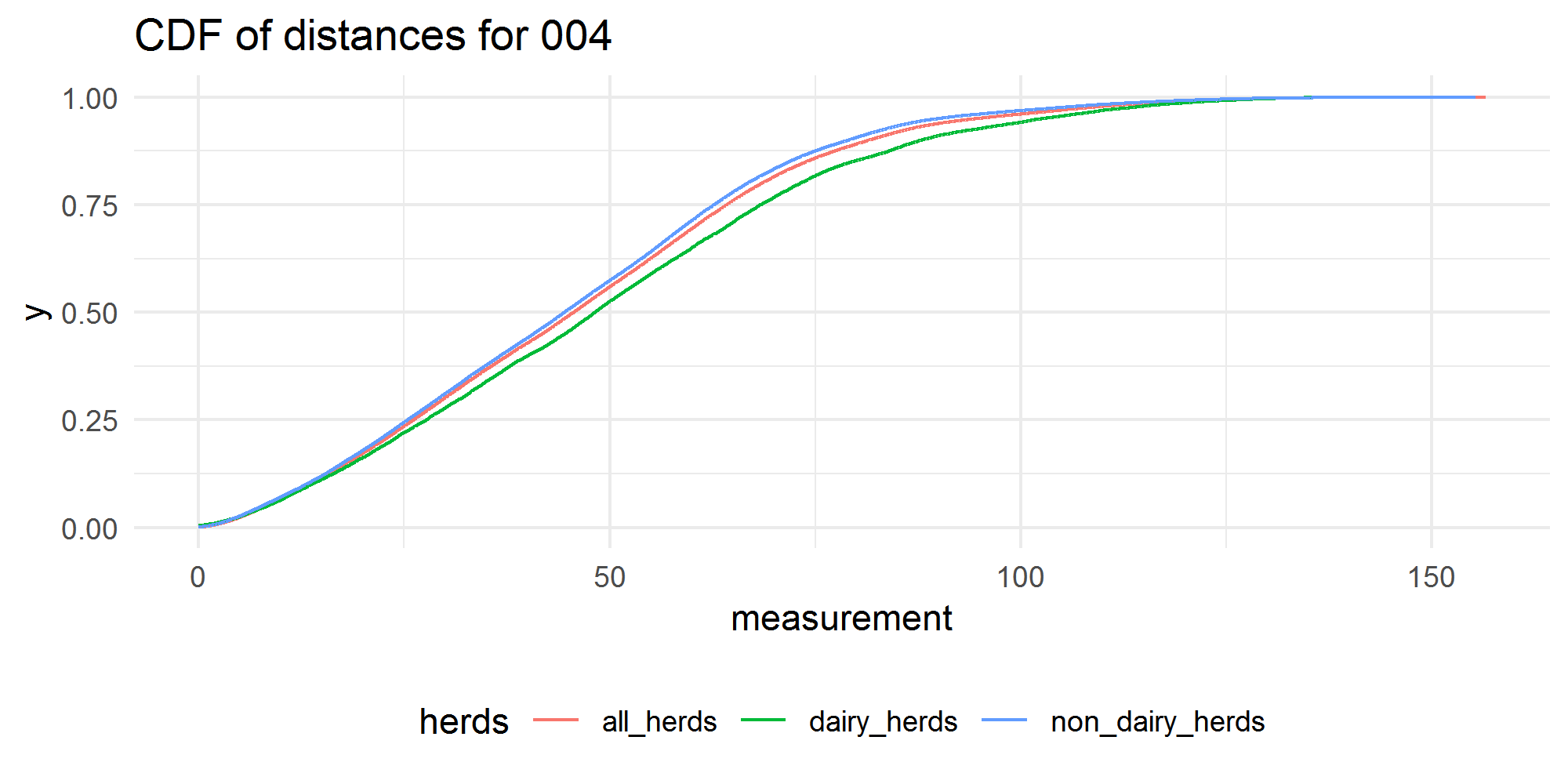
**

**Figure S15.** The Cumulative Density Function (CDF) of MLVA type 004.

**
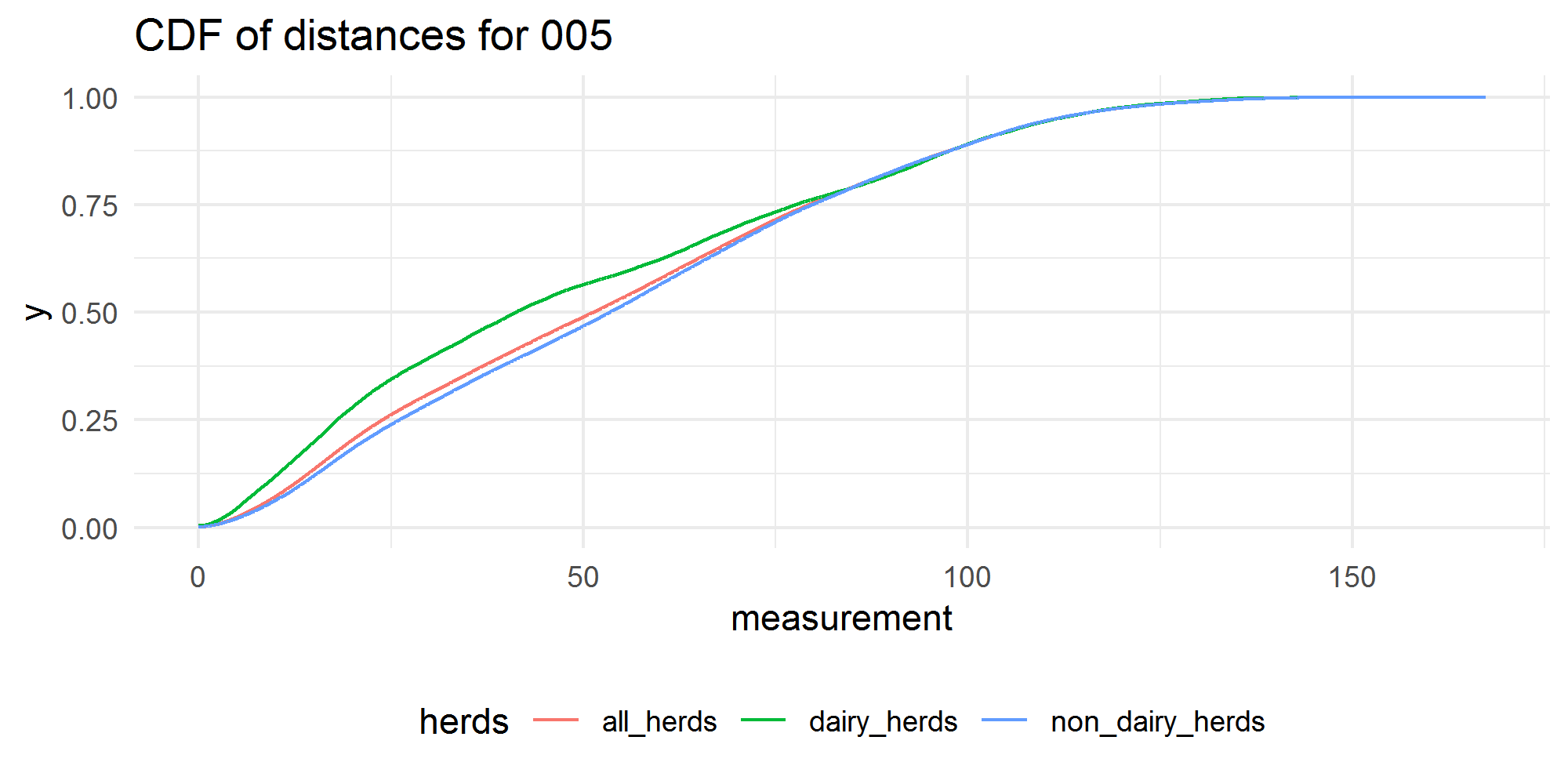
**

**Figure S16.** The Cumulative Density Function (CDF) of MLVA type 005.


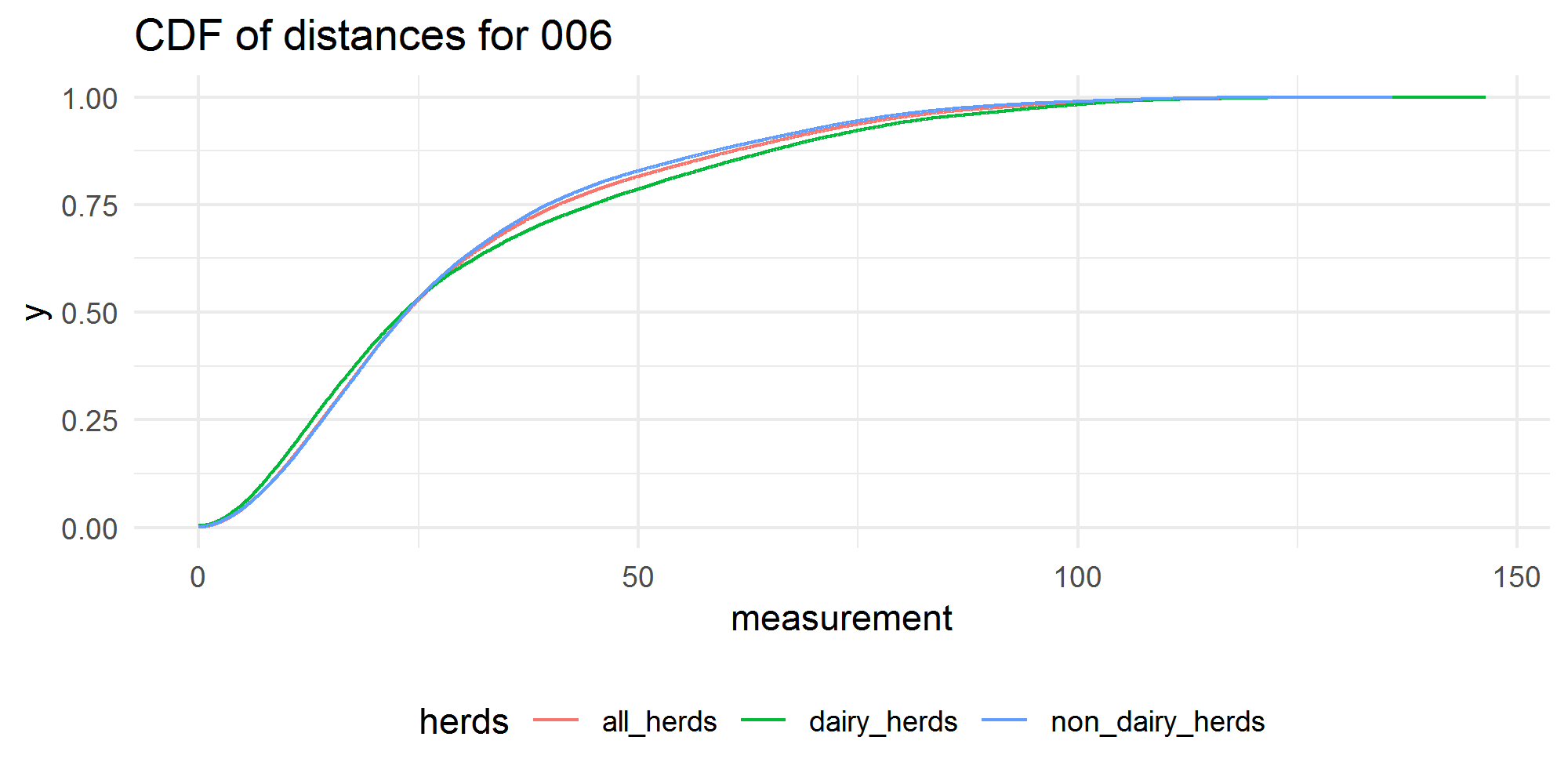


**Figure S17.** The Cumulative Density Function (CDF) of MLVA type 006.


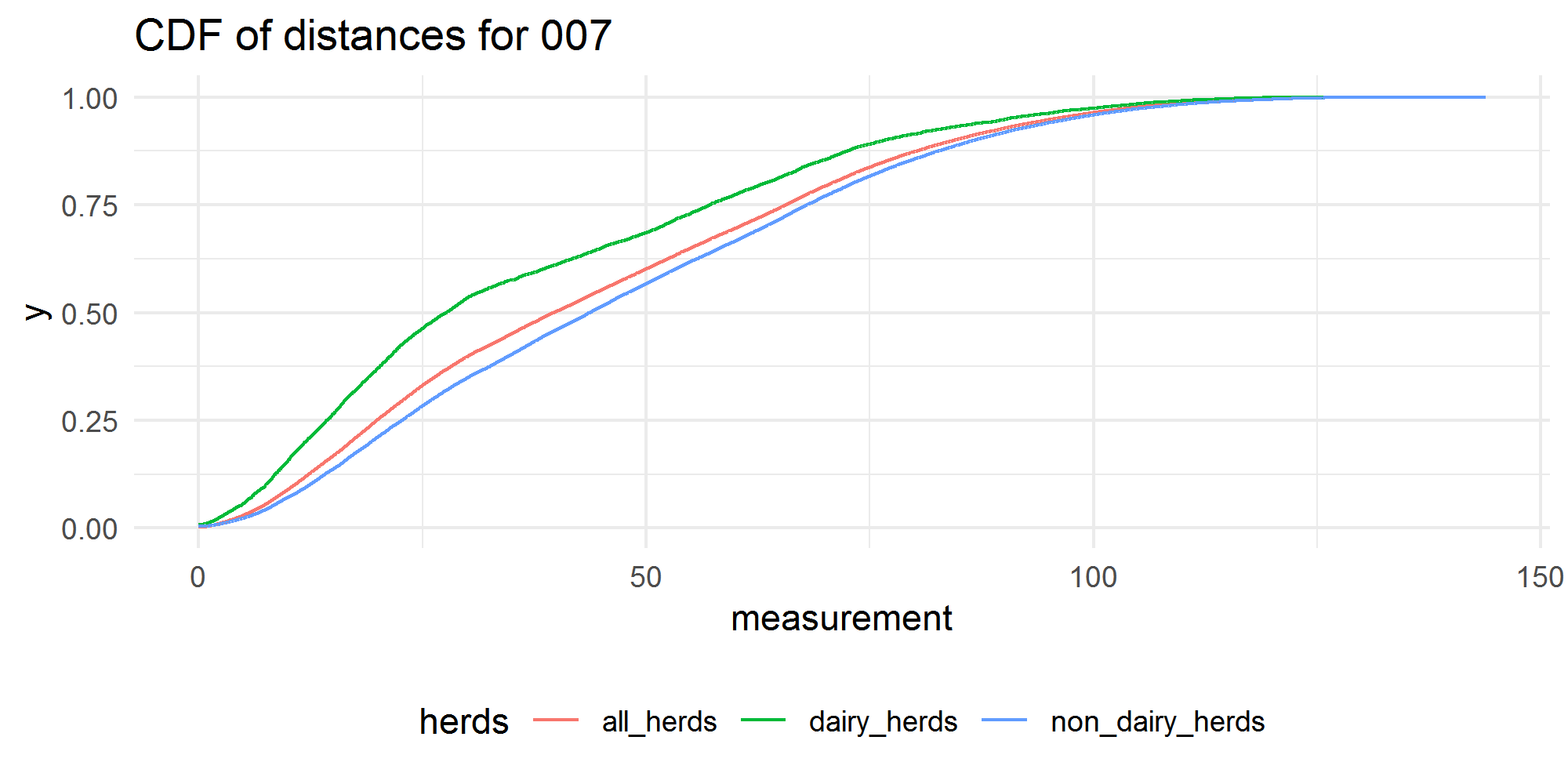


**Figure S18.** The Cumulative Density Function (CDF) of MLVA type 007.

**
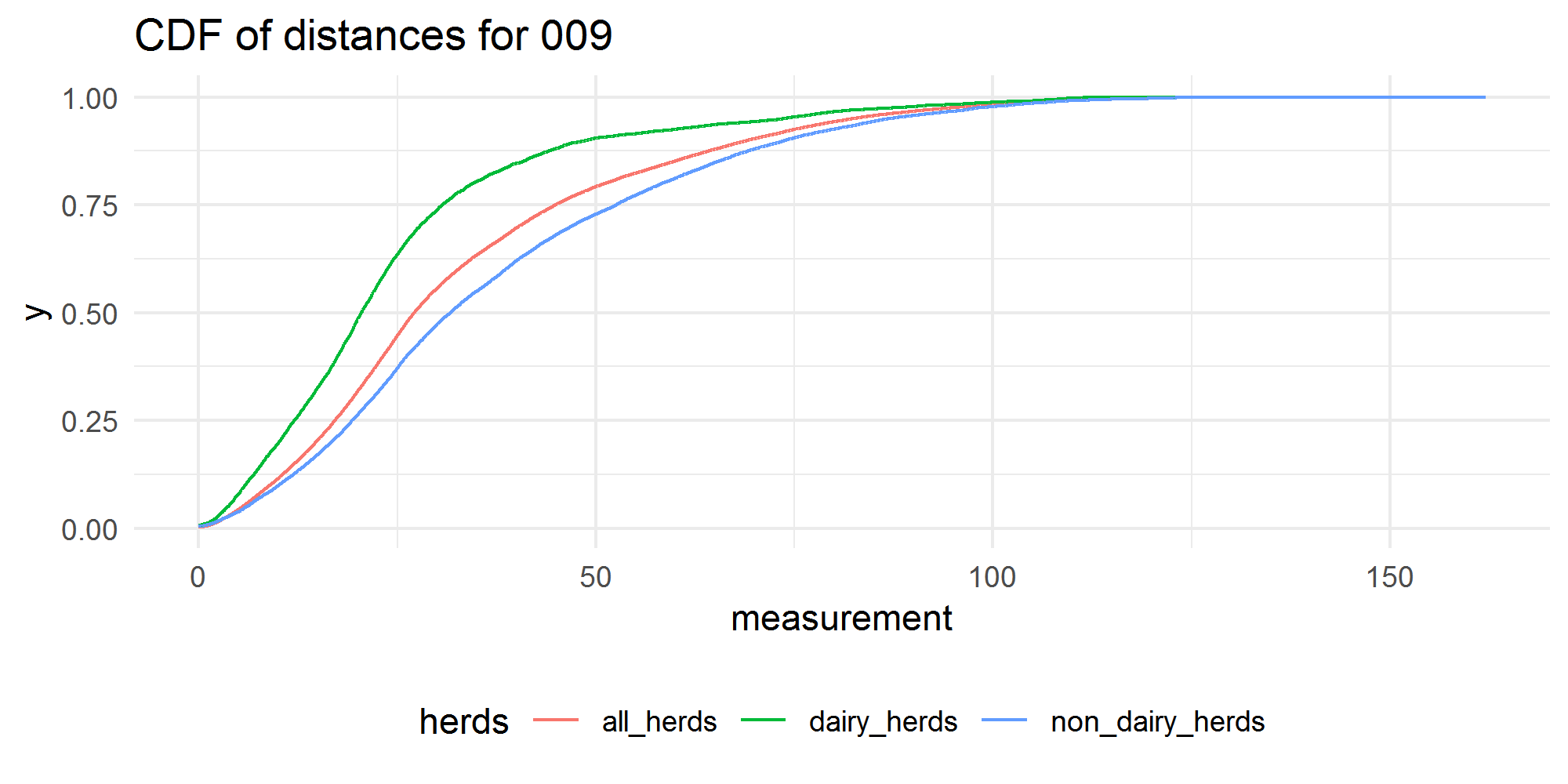
**

**Figure S19.** The Cumulative Density Function (CDF) of MLVA type 009.


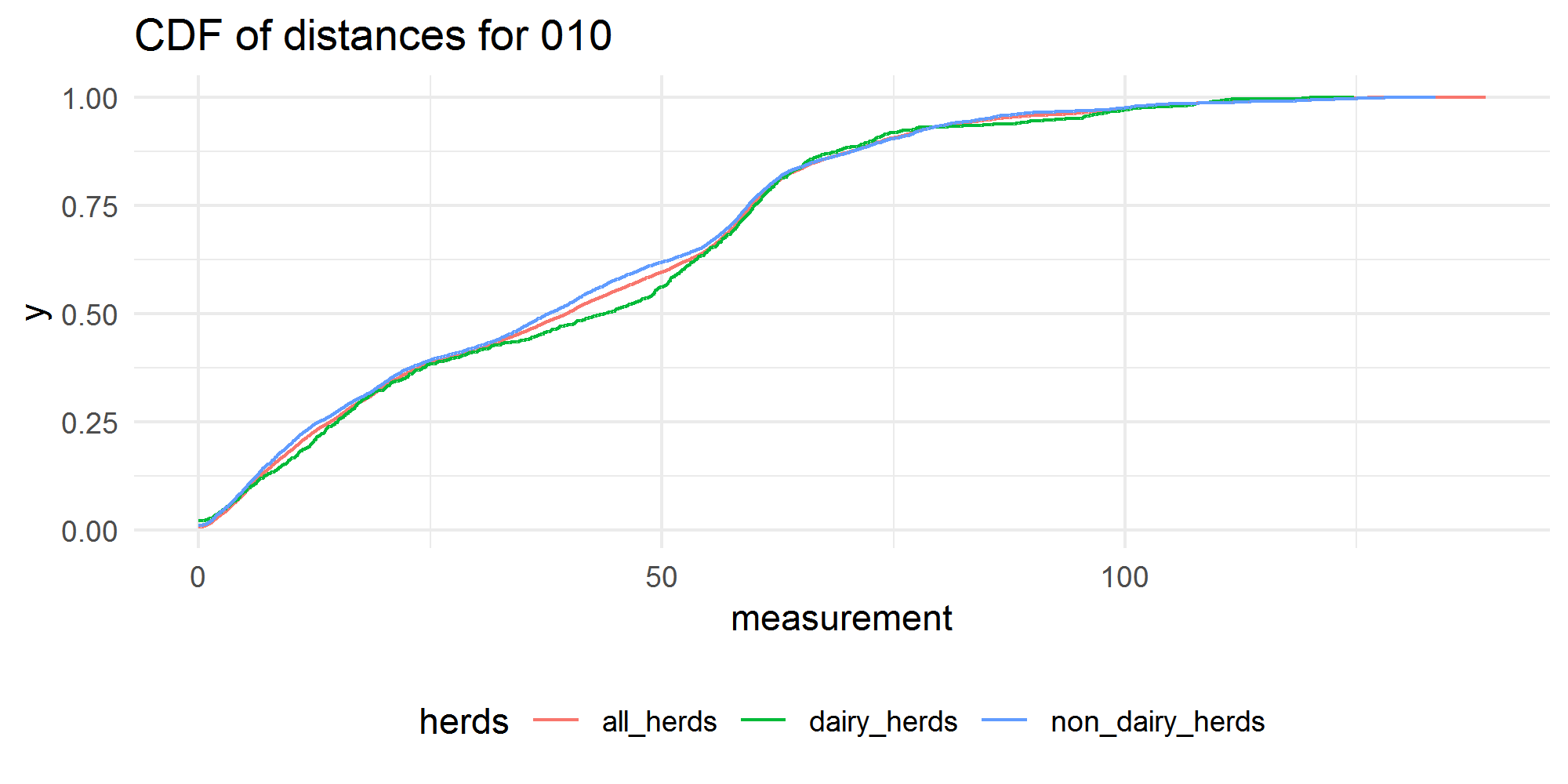


**Figure S20.** The Cumulative Density Function (CDF) of MLVA type 010.


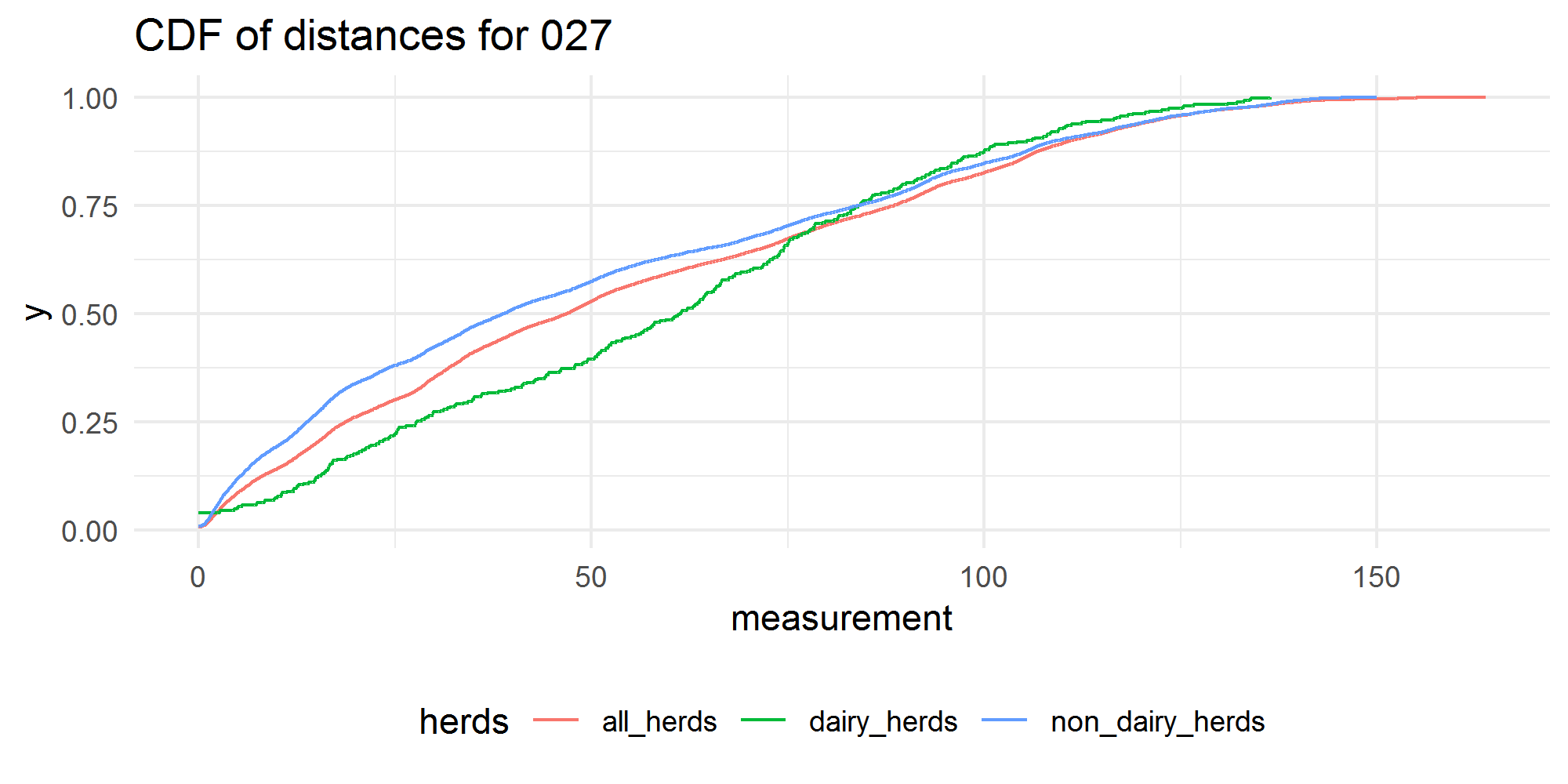


**Figure S21.** The Cumulative Density Function (CDF) of MLVA type 027.

**
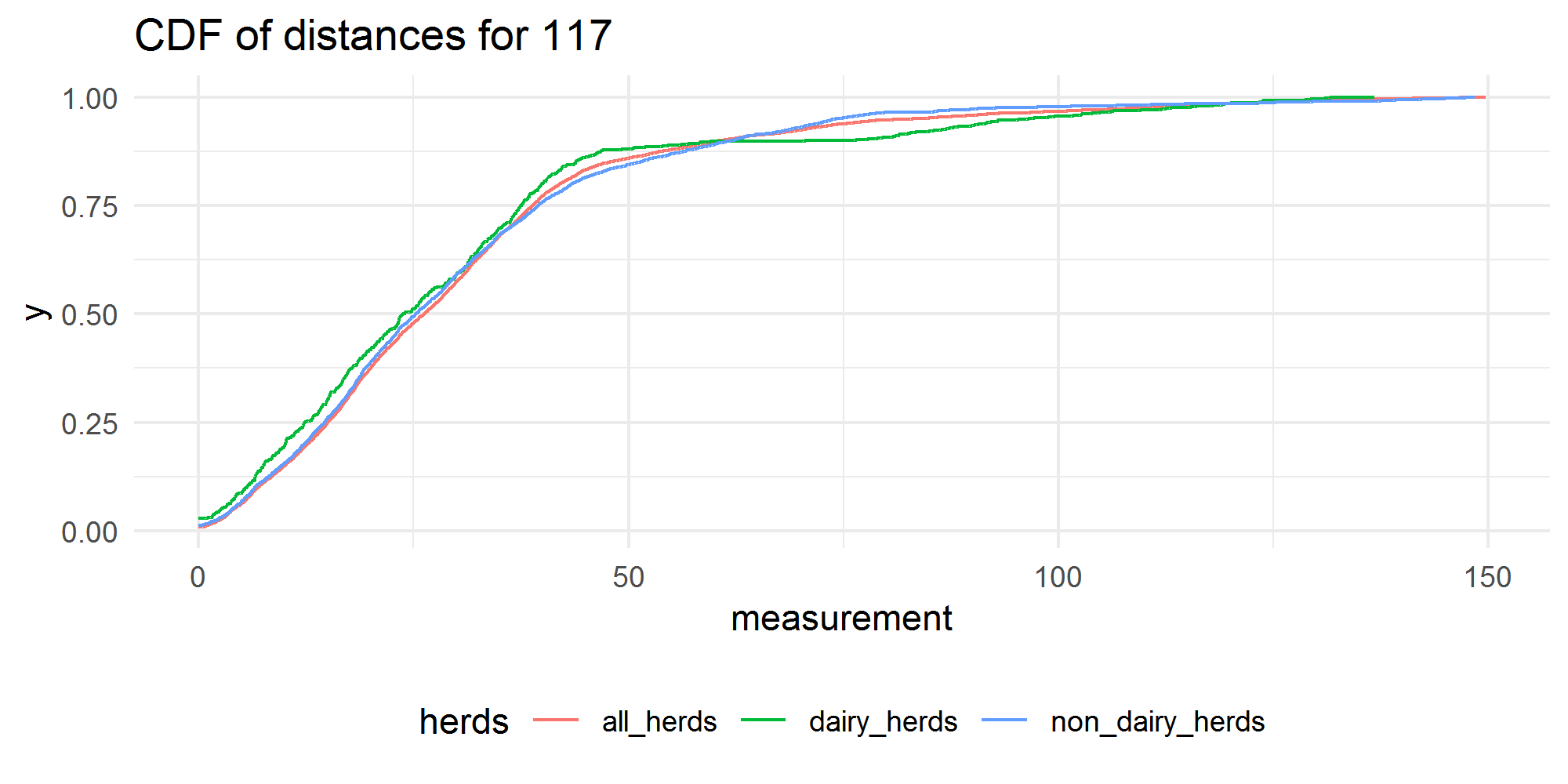
**

**Figure S22.** The Cumulative Density Function (CDF) of MLVA type 117.

**
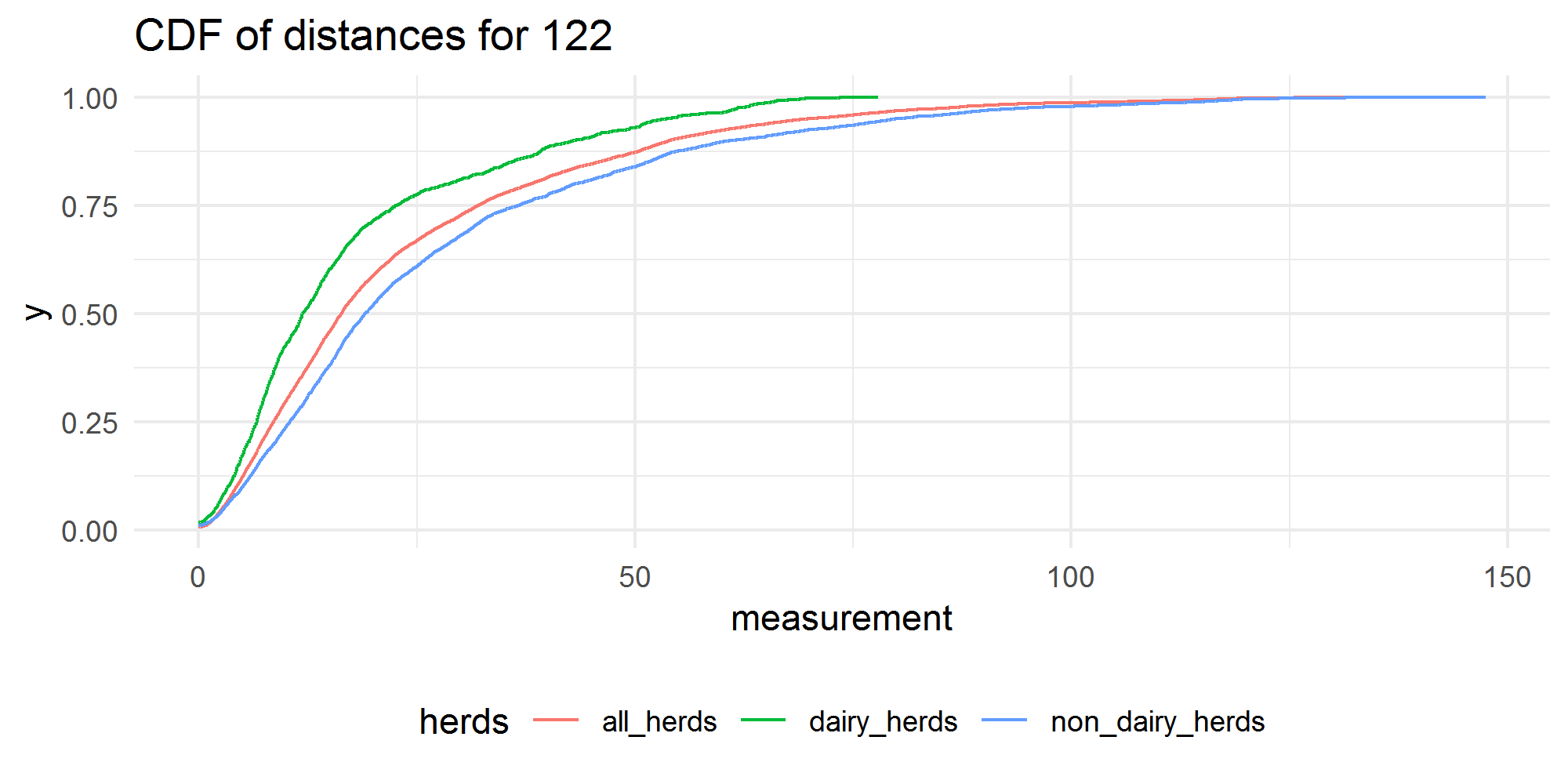
**

**Figure S23.** The Cumulative Density Function (CDF) of MLVA type 122.
